# Implicit processes do not contribute to learning to reach in small mirror reversed visuomotor environments

**DOI:** 10.1371/journal.pone.0333564

**Published:** 2026-06-08

**Authors:** Sarvenaz Heirani Moghaddam, Erin Krista Cressman, Gerome Aleandro Manson

**Affiliations:** 1 School of Human Kinetics, University of Ottawa, Ottawa, Ontario, Canada; 2 School of Kinesiology and Health Studies, Queen’s University, Kingston, Ontario, Canada; University of Giessen: Justus-Liebig-Universitat Giessen, GERMANY

## Abstract

Learning to reach with a small visuomotor rotation (VR), where visual feedback regarding hand motion is rotated relative to one’s hand trajectory, has been shown to arise unconsciously (i.e., implicitly). It is unclear whether implicit processes also support learning to reach with a small mirror reversal (MR), where visual feedback regarding hand position is reflected across the body midline. To address this gap, we asked whether implicit processes contribute to learning to reach with a small MR distortion. Forty-two right-handed participants reached to targets located 10° to the left and right of the body midline using a Kinarm exoskeleton robot. Half of the participants experienced a VR distortion (VR participants), in which cursor feedback was rotated 20° clockwise or counterclockwise relative to hand motion. The remaining participants experienced a small MR distortion (MR participants) of a similar magnitude (20°), such that cursor feedback was reflected across the body midline (y-axis). Following reaches with a VR or MR distortion, participants completed assessment trials in which they reached without cursor feedback to assess implicit learning. Analysis of angular errors (AE) revealed that all VR participants learned to reach with the VR distortion, however, only 55% of MR participants learned to reach with the MR distortion (MR-L group). AEs on the no-cursor assessment trials revealed that VR participants engaged in implicit learning. However, there was no evidence of implicit learning in the MR participants, regardless of whether they learned to reach with the MR distortion or not. These findings demonstrate that even when participants learn to reach with small MR distortions, implicit learning processes are not engaged and do not support motor learning. The absence of implicit learning when reaching with a MR distortion compared to a VR distortion suggests that MR and VR learning may be distinct forms of motor learning (e.g., skill acquisition versus motor adaptation respectively).

## Introduction

Motor learning is a broad term encompassing a range of concepts and experimental approaches [1,2]. Traditionally, motor learning has been defined as a set of processes that lead to a relatively permanent change in behaviour as a result of practice [2]. In 2019, Krakauer and colleagues broadened the scope of this definition to include both skill acquisition and skill maintenance under the umbrella term of motor learning. Skill acquisition refers to “the processes by which an individual acquires the ability to rapidly identify, select, and execute an accurate movement given a sensory stimulus and/or the current state of the body and world,” while skill maintenance, also commonly referred to as motor adaptation, refers to “the ability to maintain performance levels of existing skills under changing conditions.”

Motor learning has been studied in the laboratory by having participants reach in a novel visuomotor environment. For example, a cursor’s trajectory on the screen can be rotated 20° clockwise (CW, or rightwards) relative to hand motion (visuomotor rotation, *VR*; [3–5]). If a participant reaches directly to the target, the cursor veers slightly to the right of the target. In VR paradigms the magnitude of the cursor rotation is typically kept constant across all target locations, ensuring that the 20° CW rotation experienced remains constant regardless of target location. Thus, a participant must reach 20° counterclockwise (CCW) or left of all targets in order for the cursor to land on the target.

Another example of altered visual feedback is mirror reversed (MR) cursor feedback [6–8]. In an MR paradigm, the cursor motion on the screen is mirrored across the body midline (i.e., y-axis) relative to hand position. Thus, contrary to a VR distortion, the size and direction of the cursor distortion are dependent on a participant’s hand position relative to the body midline. If the target is placed 10° to the right of the body midline, reaching directly to the target brings the cursor 10° to the left of the body midline. In this specific example, the magnitude of the MR distortion remains constant at 20° when reaching directly to the right target. However, because cursor motion is mirrored across the body midline, any deviation in hand position will result in a change in the distortion magnitude. For example, if a participant reaches 15° to the left of the body midline, cursor feedback would be displayed 15° to the right of the body midline, introducing a 30° distortion between cursor and hand motion. In MR paradigms, the direction of the distortion also changes from CW to CCW, depending on whether the participant is reaching to the left or right of their body midline respectively.

Reaching in a VR versus an MR environment has been shown to lead to ‘aftereffects’. If participants are instructed to reach directly to the target in the absence of cursor feedback after reaching in a VR environment, they continue to reach as if the VR is still present [9–11]. These aftereffects following learning to reach with a VR distortion are proposed to arise implicitly and reflect unintentional changes that arise due to experiencing a sensory prediction error [12]. Sensory prediction errors arise due to the difference between expected and actual sensory feedback experienced [12–14]. Implicit processes have been shown to play the dominant role when one learns to reach with a VR distortion of less than 30°, even if one is made aware of the cursor distortion and/or instructed on how to counteract the VR distortion (see [15–17]). Given this implicit engagement, and in accordance with previous literature, we refer to visuomotor distortions of 30° or less as small in magnitude (see [15–17]).

In contrast to learning to reach with a VR distortion, implicit processes have not been implicated in learning to reach with a large MR distortion (i.e., visuomotor distortions greater than 40°). For example, Wang and Taylor [7] found no evidence of aftereffects immediately following reaches with a 90° MR distortion. Wilterson and Taylor [8] also observed no aftereffects following five days of reaching with a 45° MR distortion, and Telgen et al. [6] showed that reaching with a 40° MR distortion produced no aftereffects on post-test trials with aligned cursor feedback. Instead, Wilterson and Taylor [8] demonstrated that learning to reach with a 45° MR distortion engaged explicit processes (i.e., conscious strategy), as has been shown when reaching with a large VR distortion [15–17].

The role of implicit processes in learning to reach with a small MR distortion has yet to be established. Thus, we aimed to determine whether implicit processes, which are prominent in learning to reach with a small VR distortion, also play a dominant role in learning to reach with a small MR distortion. Furthermore, we compared the contributions of implicit processes to learning to reach with a small MR distortion versus a small VR distortion to contrast the learning processes engaged. We hypothesized that implicit processes would have a negligible contribution to learning to reach with a small MR distortion. Thus, the MR participants would show minimal aftereffects in the no-cursor trials following reaches with the visuomotor distortion compared to VR participants. The absence of implicit contributions to learning to reach with an MR distortion would suggest that different learning processes are engaged when reaching with an MR versus VR visuomotor distortion.

## Methods

### Participants

Forty-two participants (F = 25), aged 19–45 years (24.0 years ± 4.9 years) were recruited from the Queen’s University community and randomly divided into two groups; (1) VR group (N = 22; F = 13) and (2) MR group (N = 20; F = 10). The VR group was further divided into two subgroups of participants, including a VR counterclockwise group (CCW; VR-CCW group; N = 11; F = 6) and a VR clockwise group (CW; VR-CW group; N = 11; F = 8). All participants were naïve to the purpose of the experiment and had never participated in research involving reaches with altered visual feedback. The experimental protocol was approved by the Queen’s University Health Sciences and Affiliated Teaching Hospitals Research Ethics Board (HSREB).

Upon arrival at the laboratory, participants were provided with an overview of the experiment via a PowerPoint presentation. Participants provided written informed consent and were informed that they could withdraw at any time without penalty. Participants then completed the Edinburgh Handedness Inventory (M = 93, SD = 11; [18]), and were determined to be right-handed. Participants also completed a brief neurological questionnaire (adapted from 18), to ensure that they did not demonstrate neurological impairment.

### Experimental apparatus

Participants reached to targets using the Kinarm Exoskeleton Lab (Kinarm, Kingston, ON, Canada; [17–19]). The Kinarm was positioned adjacent to the experimenter’s computer workstation and consisted of a downward-facing computer monitor with a refresh rate of 120 hertz (Hz) and a reflective surface placed below the computer monitor. The downward-facing monitor projected visual stimuli onto the reflective surface.

Participants were seated in a height-adjustable wheelchair, and the experimenter adjusted the height and distance of the chair from the reflective surface, such that participants were able to comfortably see and reach the visual stimuli presented on the screen. Participants’ right and left arms were each placed in two size-adjustable troughs (one between the wrist and the elbow, and the other between the elbow and the shoulder). The left arm remained stationary throughout the experiment. Participants moved their right arm through flexion, extension, abduction, and adduction of the elbow and the shoulder in the horizontal plane. The position of the right index finger was represented on the screen as a cursor (i.e., white circle, 0.5 cm in diameter) and the position of this finger was recorded at a sampling rate of 1000 Hz. The reflective surface and a velcro-secured drape around the participant’s neck blocked their vision of their right limb.

### Types of trials

**Fig 1:**
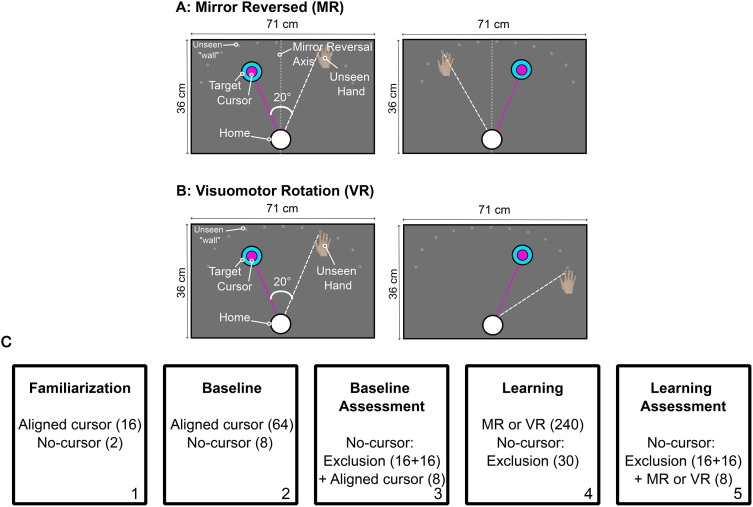
Experimental setup. **A and B:** Visual stimuli displayed during reaching trials with the visuomotor distortions. Target placement for this experiment included two targets positioned 10° to the right and left of center (i.e., 20° apart) at 10 cm from the home position. **A:** For the mirror reversed (MR) group, cursor motion was mirrored across the body midline (i.e., the y-axis) relative to the index finger motion. When the left target was shown, the index finger had to move 20° to the right of the target in order for the cursor to land on the target and when the right target was shown, the index finger had to move 20° to the left of the target in order for the cursor to land on the target. The dotted line in the middle depicts the mirror axis, which was not visible to participants. **B:** For the visuomotor rotation (VR) group, the cursor motion was rotated 20° clockwise (CW) or counterclockwise (CCW) relative to index finger motion. **C:** Overview of blocks of trials and the number of trials (in parentheses) completed within each block for each group of participants.

**Fig 2:**
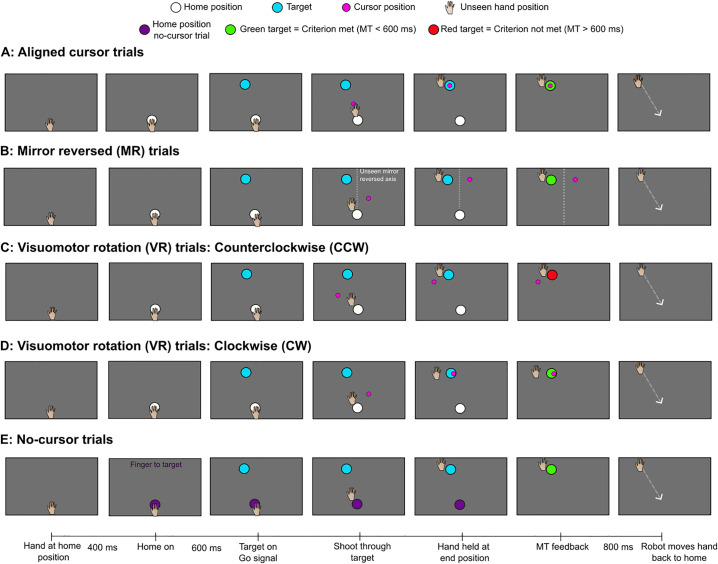
Trials completed and accompanying timeline. The hand and index finger were hidden from participants’ view. **A.** Aligned cursor trials: Reaches with aligned cursor feedback. The cursor motion was aligned to a participant’s index finger’s trajectory and was continuously visible throughout the reach until the finger crossed the target array. **B** MR trials: Reaches with mirror reversed cursor feedback. The cursor’s trajectory was mirrored across the body midline relative to the trajectory of the index finger and was visible throughout the reach until the finger crossed the target array. **C-D** VR trials: Reaches with rotated cursor feedback. The cursor’s trajectory was rotated 20° CCW (**C**) or 20° CW (**D**) relative to the trajectory of the index finger and was visible throughout the reach until the finger crossed the target array. **E.** No-cursor trials: Participants were instructed to reach to the target. Movement time (MT) feedback was provided at the end of each movement, before the robot moved the hand back to the home position to begin the next trial via a target colour change (green = MT criterion met; red = MT criterion not met).

Fig 1C provides an overview of the block structure within the experiment. The blocks of trials included familiarization, baseline, baseline assessment, learning and learning assessment. A detailed description of the trial types completed within each block is provided in the next section and illustrated in Fig 2.

### Aligned cursor trials

Participants made “shooting” movements towards two targets 10 cm from the home position. The targets were blue circles (0.75 cm in diameter) that appeared 10° to the right and left of the y-axis, which was aligned with a participant’s midline (Fig 1A and 1B). The two targets appeared with equal probability. In aligned cursor trials, participants reached while a white cursor (0.5 cm in diameter) accurately represented their index finger position on the screen (Fig 2A).

Each trial began with participants holding their index finger at the home position for 400 ms. The home position and the cursor then appeared. Following another 600 ms, one target appeared. Participants were instructed to move the cursor through the target as quickly and accurately as possible by performing a slicing action. This slicing action was completed as a ballistic shooting movement such that a participant’s index finger passed through the target at approximately peak velocity [20]. Movements were terminated by a soft wall placed 2 cm beyond the target. This soft wall (spring constant 150 N/m) made the movement feel more natural and encouraged participants to move rapidly through the target. Visual feedback regarding the right index finger position was available up until the participant’s index finger moved 10 cm outwards from the home position and crossed the target array. At this time, the cursor stopped, and participants no longer received visual feedback regarding their index finger’s position. Movement onset was defined online as the time when the centre of the cursor moved 0.5 cm away from the centre of the home position and when velocity first increased above 0.03 m/s and remained above 0.03 m/s for the subsequent 9 ms. Movement end was defined as the time that the hand crossed the target array. Movement time (MT) was defined as the time between movement onsent and movement end.

Participants received visual feedback regarding their MT in the form of a target colour change once their index finger reached the soft wall. The goal MT was less than 600 ms, such that the target turned green if MT was less than 600 ms and red if MT was greater than 600 ms. On all trials, the hand remained in the movement end location for 800 ms before the robot moved the participant’s index finger back to the home position along a linear path in a MT of 1000 ms in the absence of cursor feedback to start the next trial (see Fig 2). If participants attempted to move out of the linear path, an equal and opposite force was applied to their hand to keep the hand within the linear path.

### VR and MR trials

These trials were similar to aligned cursor trials but participants experienced a VR (Fig 2C and 2D; VR group) or MR (Fig 2B; MR group) cursor distortion. For the VR distortion, visual feedback of the index finger motion was rotated 20° CW (VR-CW group) or CCW (VR-CCW group) relative to hand motion. For the MR group, visual feedback of the hand position was mirrored across the body midline. As a result, reaching directly to the right target (10° right of body midline) resulted in the cursor appearing 10° left of body midline, producing a 20° distortion between the cursor and hand motion. Participants were not informed about the presence of the visuomotor distortion and were instructed only to move the cursor through the target as quickly and accurately as possible.

### No-cursor trials: Exclusion trials

No-cursor trials were similar to aligned cursor trials except that the cursor remained off during the movement (Fig 2E). These trials were used to establish implicit processes (i.e., aftereffects) in accordance with the Process Dissociation Procedure (PDP; adapted from Werner et al., [17]). At the beginning of the these no-cursor exclusion trials, participants were instructed:

*“You are now going to reach when you cannot see your* index finger*, as there will be no-cursor on the screen. Do not use anything you may have learned to get the cursor to the target. Instead, aim so that your* index finger *goes straight through the target as you did during baseline reaches.”*

The absence of the cursor in no-cursor trials was cued with a change in colour of the home position, such that on these trials the home position was purple and the instruction “Finger to target” appeared on the screen above the target.

In the familiarization, baseline and learning blocks, one no-cursor trial was completed for every 8 cursor trials. In the baseline block, participants completed 64 aligned cursor reaches and 8 no-cursor reaches. In the learning block, participants reached with either a VR or MR cursor distortion for 240 trials and completed 30 no-cursor trials. In the baseline and learning assessment blocks, participants completed 16 no-cursor exclusion trials, followed by 8 reaching trials with cursor feedback and then an additional 16 no-cursor exclusion trials. The 8 cursor trials were included in the assessment blocks to ensure that any learning was maintained.

### Data analyses

All reaching trials were analyzed using custom-written MATLAB scripts (MATLAB R2022a, The MathWorks, Inc.). Hand angular error at movement endpoint (AE), defined as the angular difference between a reference vector connecting the home position to the target and a reach vector connecting the home position to the finger position at the end of the movement, was the main variable of interest. RT and MT were also analyzed. RT was defined as the time between target onset and movement start (defined as the time when the centre of the cursor moved 0.5 cm away from the centre of the home position and velocity first increased above 0.03 m/s and remained above 0.03 m/s for the subsequent 9 ms). MT was defined as the time from movement start until movement end (defined as the time that the index finger crossed the target array).

### Outlier procedures

The following variables were used to screen for outliers: Start X and Start Y (x and y coordinates of the movement’s start position), AE and MT. If any of these variables was greater than 3 standard deviations (SD) from the participant’s average on a specific type of trial within each block (baseline, baseline assessment, learning, or learning assessment), the trial was removed from further analyses. For example, the AE of an aligned cursor trial was compared to the mean AE of the aligned cursor reaches within that block, while the AE of a no-cursor trial was compared to the mean AE of the no-cursor trials within the same block. Any reach with a MT greater than 600 ms or less than 100 ms was also flagged as an outlier and removed from analyses. Across all groups, participants completed 14,226 trials. 926 (6.51%) of these trials were classified as outliers and removed from the analyses presented below.

### Learning

A subset of reaches with cursor feedback in the baseline and learning blocks were designated as early and late trials. The first 24 trials were designated as early trials and the last 24 trials as late trials. Each participant’s data were screened to determine if they demonstrated learning by examining individual learning curves and comparing a participant’s average AE in the late learning phase (last 24 trials) with their AE in the late baseline phase (last 24 trials) for both right and left targets. To confirm learning, participants had to show a difference in AE in the late learning phase compared to the late baseline phase that was greater than the mean AE in the late baseline phase plus 3 standard deviations and reach in the correct direction. Based on our learning criteria, we found that all VR participants learned, while only 11 participants in the MR group demonstrated learning. Participants who reached with the MR distortion and met the learning criteria were placed in the MR-learner group (MR-L). Participants who did not meet this criterion formed the MR-non-learner (MR-NL) group. For all participants, early and late AE data for each target in the learning block were nomalized by subtracting early and late AE in the baseline block, respectively.

The absolute values of the normalized AEs were compared between groups in a 4 group (MR-learner (MR-L), MR-non-learner (MR-NL), VR-CW, VR-CCW) x 2 target (right, left) x 2 time (early trials, late trials) mixed analysis of variance (ANOVA) with repeated measures (RM) on the last two factors. Participants who trained with VR CW and CCW rotations were considered to form separate groups to preserve target-wise compatibility with MR participants and to maintain comparable sample sizes across groups. We also compared AE variability and mean RT in the learning block across groups in a 4 group (MR-L, MR-NL, VR-CW, VR-CCW) x 2 target (right, left) x 2 time (early trials, late trials) mixed ANOVA with RM on the last two factors. AE variability for each participant was calculated as the standard deviation of AEs across the early or late learning trials for each target. To confirm that participants across all groups complied with the MT criteria, mean MT was analyzed in a 4 group (MR-L, MR-NL, VR-CW, VR-CCW) × 2 target (right, left) × 2 time (early trials, late trials) mixed ANOVA with RM on the last two factors.

### Implicit learning

An implicit index was calculated for each target within the baseline and learning assessment blocks. The implicit index was defined as the mean AE of the no-cursor trials:

1) Implicit index = ${\overset{―}{x}}_{AEofno-cursortrials}$

These indices were then used to calculate implicit learning for each target according to the following formula:

2) Implicit learning = Implicit index (learning assessment) – Implicit index (baseline assessment)

To allow for meaningful comparisons across groups and targets, all AE values were normalized so that positive values reflected movement in the expected direction of learning, regardless of target.

Implicit learning for the right and left targets were compared across groups in a 4 group (MR-L, MR-NL, VR-CW, VR-CCW) x 2 target (right, left) mixed ANOVA with RM on the last factor. We also compared the magnitude of implicit learning to zero for each group by performing a Bayesian one-sample *t*-test. These *t-*tests allowed us to establish the presence of implicit learning in the different groups.

All analyses were completed using JASP, RStudio and Microsoft excel softwares. In the case where the test of homogeneity of variance (Levene’s test) was violated, a Welch correction was performed. Further, if the assumption of sphericity was violated, Greenhouse-Geisser corrected degrees of freedom were reported. The significance threshold for all statistical tests was set at *p* < 0.05 and post hoc tests with Bonferroni corrections for multiple comparisons were used to find the locus of significant effects or interactions. For clarity, only the breakdown of the highest-order significant interaction is reported. Data are reported as group means and standard deviations, reflecting between-participant variability.

## Results

### Learning

**Fig 3:**
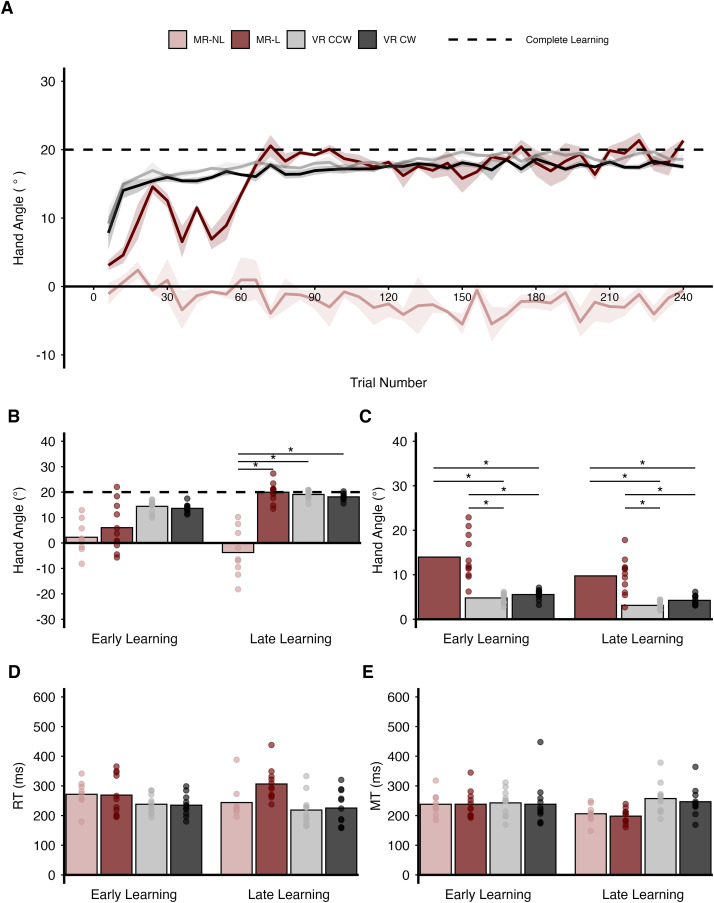
A: Learning curves for each group across trials. Endpoint angular errors (AE) in degrees are averaged over three consecutive trials in the learning block. The shaded areas represent the standard error of the mean. **B**: Absolute values corresponding to average AE during early and late learning trials for each group. In A and B, dashed line at y = 20 indicates reach adjustments demonstrating complete learning. **C**: Average variability of AEs during early and late learning trials for each group. **D**: Average RT and **E**: average MT during early and late learning trials for each group. The MR-non-learner (MR-NL) group is shown in light pink, MR-learner (MR-L) group in maroon, the VR-CCW group in gray, and the VR-CW group in black. Individual participant data are represented as circles. Asterisks denote significant group differences (*p* < 0.05).

With respect to learning, ANOVA revealed significant main effects of group (*F*(3,38) = 39.245, *p* < 0.001, *η*_*p*_*²* = 0.756) and time (*F*(1,38) = 14.913, *p* < 0.001, *η*_*p*_*²* = 0.282), as well as a significant group × time interaction (*F*(3,38) = 12.828, *p* < 0.001, *η*_*p*_*²* = 0.503) and group × time × target interaction (*F*(3,38) = 5.359, *p* = 0.004, *η*_*p*_*²* = 0.297). Post hoc analysis indicated that early mean AE for the MR-L group (right target: M = 3.48° ± 12.85°, left target: M = 8.56° ± 14.20°) was not different from early mean AE for the MR-NL group (right target: M = 9.04° ± 12.60°, left target: M = −4.95° ± 11.19°) or both the VR-CW (right target: M = 12.28° ± 3.04°, left target: M = 14.94° ± 3.22°), and VR-CCW groups (right target: M = 13.10° ± 2.74°, left target: M = 15.72° ± 2.78°) at both targets (all *p* > 0.05). Mean AE in the late learning trials for the MR-L group (right targe: M = 20.29° ± 3.04°, left target: M = 19.64° ± 7.24°) was significantly greater than their mean AE in the early learning trials at both targets (both *p* < 0.05), demonstrating changes in reaches. In contrast, the MR-NL group did not show the same increase in mean AE from early to late learning trials (late mean AE for MR-NL: right target: M = −7.14° ± 13.94°, left target: M = −0.39° ± 18.25°; *p* = 0.454). There were no differences in AE between early and late learning trials for either VR group (Late VR-CCW right target: M = 17.98° ± 2.14°, left target: M = 20.16° ± 2.15°; late VR-CW right target: M = 18.50° ± 1.61°, left target: M = 17.76° ± 2.47°; *p* > 0.05), and the magnitude of mean AE in the late learning trials did not differ significantly between the MR-L group and either VR group (both *p* > 0.05; Fig 3A and 3B).

Analysis of AE variability revealed significant main effects of group (*F*(3,38) = 56.031, *p* < 0.001, *η*_*p*_*²*  = 0.816), target (*F*(1,38) = 14.280, *p* < 0.001, *η*_*p*_*²* = 0.273), time (*F*(1,38) = 10.449, *p* = 0.003, *η*_*p*_*²* = 0.216), and a significant group x target interaction (*F*(3,38) = 4.381, *p* = 0.010, *η*_*p*_*²* = 0.257). Post hoc analysis revealed that the MR-L group (early: M = 14.0° ± 5.2°; late: M = 9.7° ± 4.2°) and the MR-NL group (early: M = 14.3° ± 5.2°; late: M = 10.8° ± 4.0°) demonstrated similar AE variability (*p* = 1.000), while AE variability was significantly greater in both the MR-L and MR-NL groups than the VR-CW (early: M = 5.6° ± 1.1°; late: M = 4.2° ± 1.0°) and the VR-CCW groups (early: M = 4.8° ± 1.1°; late: M = 3.1° ± 0.8°; all *p* < 0.001). Across all groups, AE variability was significantly lower in late learning trials compared to early learning trials (*p* < 0.001). Post hoc analysis of the group x target interaction revealed that AE variability was larger for reaches towards the left target compared to the right target only for the MR-NL group (*p* < 0.001).

Analysis of mean RT in the learning block revealed a significant main effect of group (*F*(3,38) = 5.249, *p* = 0.004, *η*_*p*_*²* = 0.293). Post hoc analysis revealed that the MR-L group took longer to initiate their reaches compared to the VR-CW and VR-CCW groups (all *p* < 0.05). No differences were found between the MR-L (early: M = 296.1 ms ± 67.3 ms; late: M = 306.4 ms ± 54.7 ms) and MR-NL groups (early: M = 271.8 ms ± 45.0 ms; late: M = 243.9 ms ± 57.9 ms) or the MR-NL and the VR-CW (early: M = 235.0 ms ± 36.0 ms; late: M = 225.3 ms ± 58.9 ms) and VR-CCW groups (early: M = 238.2 ms ± 31.3 ms; late: M = 218.6 ms ± 53.6 ms; all *p* > 0.05; Fig 3D).

MT data confirmed that all participants adhered to the MT criteria, completing their reaches with MTs ranging from 191 ms to 264 ms throughout the learning block. ANOVA revealed no significant differences between groups (*F*(3,38) = 1.555, *p* = 0.216, *η*_*p*_*²*  = 0.109). However, there was a significant main effect of target (*F*(1,38) = 10.215, *p* = 0.003, *η*_*p*_*²*  = 0.212) and a significant group x time interaction (*F*(1,38) = 2.917, *p* = 0.047, *η*_*p*_*²*  = 0.187). Post hoc analyses revealed that reaches to the right target were approximately 11.6 ms faster than those to the left target but no significant effects associated with the group × time interaction were observed. These results confirm that participants executed their movements in a similar time across groups (MR-L early: M = 238.2 ms ± 46.1 ms; late: M = 198.1 ms ± 24.4 ms; MR-NL early: M = 238.1 ms ± 38.9 ms; late: M = 206.1 ms ± 30.0 ms; VR-CW: early: M = 238.2 ms ± 77.4 ms; late: M = 246.7 ms ± 49.2 ms; VR-CCW: early: M = 242.8 ms ± 44.0 ms; late: M = 257.3 ms ± 53.1 ms) and within the prescribed MT constraints (Fig 3E).

### Implicit learning

Analysis of implicit learning revealed a main effect of group (*F*(3,38) = 21.006, *p* < 0.001, *η*_*p*_*²* = 0.624). Post hoc analysis indicated that the extent of implicit learning was significantly less for the MR-L group (M = 1.4°, SD = 8.6°) and the MR-NL group (M = −0.3°, SD = 8.2°) compared to the VR-CW (M = 16.7°, SD = 2.7°, both *p* < 0.001) and VR-CCW groups (M = 14.8°, SD = 3.2°, both *p* < 0.001). The magnitude of implicit learning in the VR-CW group was not different from the VR-CCW group (*p* = 0.702).

**Fig 4:**
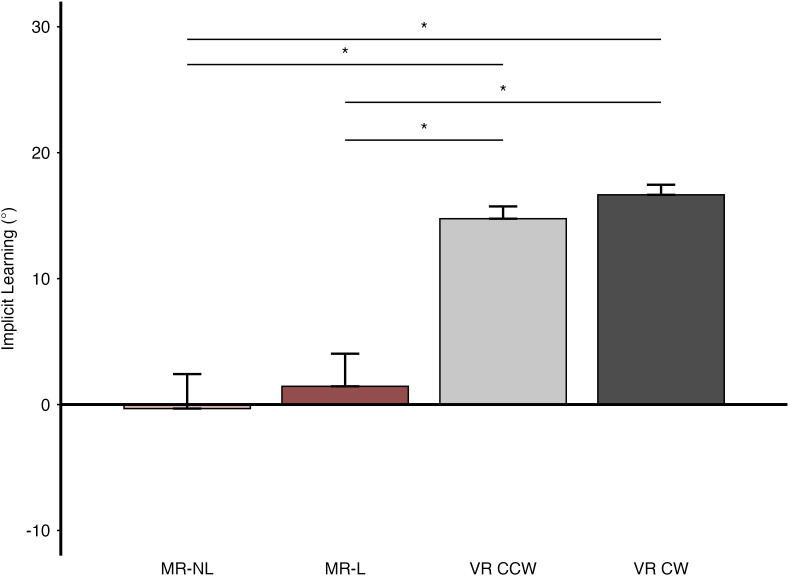
Mean implicit learning for all groups. The MR-non-learner (MR-NL) group is shown in light pink, MR-learner (MR-L) group in maroon, the VR-CCW group in gray, and the VR-CW group in black. Error bars reflect standard error of the mean. Asterisks denote significant group differences (*p* < 0.05).

Bayesian one sample *t*-tests indicated anecdotal-to-moderate support for the null hypothesis [21] in both the MR-L group and the MR-NL group (all *p* > 0.05; MR-L: *BF₁₀* = 0.340; MR-NL: *BF₁₀* = 0.324), suggesting that implicit learning was not significantly different from zero in either the MR-L or the MR-NL groups. In contrast, VR-CW and VR-CCW groups showed significant implicit learning (all *p* < 0.001; VR-CW: *BF₁₀* = 5.445 x 10^6^; VR-CCW: *BF₁₀* = 338426.099), indicating strong evidence in favor of a true effect (Fig 4).

## Discussion

This study investigated the contribution of implicit processes to learning to reach with a small MR distortion. The contribution of implicit processes to learning was directly compared following reaches with an MR versus a VR distortion. All VR participants successfully learned to reach with their distortion (CW or CCW cursor rotation). In contrast, only 11 out of 20 MR participants learned to reach with the MR distortion. The MR-L group and both VR groups exhibited similar levels of learning by the end of the learning block. Learning in the VR groups was supported by implicit processes. Importantly, MR participants did not show evidence of implicit learning, regardless of whether they learned to reach with the distortion or not.

To assess implicit learning, we measured persistent reach adjustments during no-cursor exclusion trials (i.e., aftereffects), in which participants were instructed to aim directly at the target without applying what they had learned. This method is part of the Process Dissociation Procedure (PDP; 15), which has been used previously to measure implicit processes in visuomotor learning studies following reaches with a cursor rotation [15–17,22–24]. In the current study, the VR groups demonstrated robust aftereffects of approximately 15°, confirming that implicit processes contributed to learning to reach with a VR distortion. Specifically, the magnitude of aftereffects observed in the VR groups in the present study is consistent with previous reports (see [25,26]. When learning to reach with a VR distortion, the magnitude of implicit learning is relatively stable, saturating at approximately 15–25° regardless of cursor rotation size [15–17,22,25]. These implicit processes are proposed to be driven by a sensory prediction error, arising due to the discrepancy between the expected sensory consequences of the movement and the actual sensory consequences of the movement experienced [12,13].

In contrast to the VR group, we found no evidence of aftereffects in either the MR-NL or MR-L groups. The lack of aftereffects suggests that reaching with an MR distortion did not engage implicit processes, regardless of whether participants learned to reach with the MR distortion. These findings are in accordance with previous research that has employed larger MR distortions [7,8,27]. Prior research has typically involved large MR distortions (i.e., 45° or greater), in which explicit processes are expected to dominate, in accordance with the VR literature [15–17,24]. Specifically, studies of VR learning have confirmed that when the rotation size increases above 30°, explicit processes are engaged alongside implicit processes. Despite the reduced distortion size in the current study, our results are consistent with studies employing larger MR perturbations [6–8]. That is, participants in the MR group (both MR-L and MR-NL participants) demonstrated no implicit learning when visual feedback was removed.

Although sensory prediction errors would likely still arise during MR reaches, the structure of these errors differs from those experienced during VR learning. In a VR task, the discrepancy between expected and actual visual feedback experienced remains constant across the workspace and movement trajectory, providing a stable signal to drive implicit learning. In contrast, an MR distortion produces a state-dependent transformation, in which the magnitude and direction of the error signal depend on the hand position relative to the mirror axis (i.e., body midline). This results in an error signal that changes within and across trials depending on reaching direction relative to the body midline, potentially limiting the ability of a sensory prediction error to drive implicit learning. Prior work has shown that when error direction fluctuates, overall learning is reduced [28] and implicit processes are diminished [29]. One explanation is that the motor system integrates a running history of experienced errors, weighting new ones relative to this record [30,31]. In the MR task, participants’ movements would generate inconsistent error signals that likely limited the potential engagement of implicit learning.

In the current study, the absence of implicit learning was observed alongside longer RTs when participants learned to reach with the MR distortion compared to the VR distortion. Previous research has observed increased RTs during tasks which place greater cognitive or planning demands on the motor system, including situations in which explicit strategies may be engaged during motor learning [26,32–34]. Haith et al. [33] directly demonstrated the link between preparation time and the engagement of explicit strategies by manipulating RT while participants reached with a 45° VR distortion. They found that when preparation time was long, explicit strategies were engaged, whereas restricted preparation times constrained learning largely to implicit processes. For small cursor rotations, where explicit contributions have been shown to be minimal [15–17], one would expect little to no effect of preparation time on learning. Consistent with this reasoning, RTs are typically not elevated during learning to reach with small VR distortions, whereas they are elevated during MR learning compared to VR learning [6,25]. The longer RTs observed in our MR-L group, together with the absence of implicit learning, are consistent with the notion that learning to reach with an MR distortion places greater cognitive and planning demands on the motor system comparted to VR learning, and that these demands may involve the engagement of explicit strategies. To note, our MR-NL participants, who did not learn to reach with the MR distortion, initiated their movements with a similar RT to VR participants.

The differential engagement of implicit processes and planning time when learning to reach with a VR versus an MR distortion suggests differences in how VR and MR distortions are learned. As suggested by Gastrock et al. [27] and Telgen et al. [6], learning to reach with an MR distortion may require the formation of an entirely new sensorimotor mapping (e.g., a novel control policy), rather than a modification of an existing sensorimotor mapping as done in visuomotor adaptation, regardless of MR distortion size. In alignment with this hypothesis, the increased AE variability across early and late learning trials in our study may reflect an evolving or unstable sensorimotor mapping as participants attempted to construct a novel control policy to compensate for the mirrored cursor feedback. It has been proposed that motor variability can serve as a mechanism for exploration, enabling the motor system to sample different movement strategies in response to novel or uncertain task demands [35]. The increased movement variability observed in MR participants compared to VR participants may be a signature of the motor system’s attempt to explore and establish a new sensorimotor mapping under conditions where implicit learning mechanisms are not engaged. This increased variability was observed across both early and late learning trials (i.e., the standard deviation of the first 24 (early) and last 24 (late) learning trials), even though mean AE did not differ significantly between the MR-L and VR groups. We interpret our variability estimates during early learning cautiously, as participants were learning to reach with the MR or VR distortion and AE would be expected to change across trials. However, the sustained increase in variability even in late learning trials suggests performance by MR participants did not stabilize to the same extent as VR participants, and participants continued to adjust their reaches from trial to trial.

Despite exploration, not all participants in the MR group were able to learn to reach with the MR distortion. A large proportion of individuals that reached with the MR distortion (MR-NL group: n = 9 of 20 participants) did not learn to reach in the MR environment. This contrasts with participants in the VR group, who all learned to reach with rotated cursor feedback. The presence of non-learners exclusively when participants reached with the MR distortion provides evidence that the MR task was more difficult to learn compared to the VR task.

To cope with task complexity and achieve some level of performance, several MR-NL participants appeared to adjust their movements to one target in the correct direction. This pattern suggests that the increased complexity of the MR distortion relative to the VR distortion may promote partial or target-specific solutions to emerge before an effective control policy is established. It is also possible that the absence of learning in some participants reflects the limited duration of training provided within a single experimental session. Prior work has shown that learning to reach with a large MR distortion may require extended practice compared to learning to reach with a visuomotor rotation [6]. However, even with extended training across multiple days, implicit processes do not appear to meaningfully contribute to reducing errors during MR learning [8], suggesting that improvements in MR performance do not rely on implicit processes even after 5 days of reaches.

In summary, our findings demonstrate that implicit processes support learning to reach with a VR distortion but do not contribute to learning to reach with an MR distortion, even under conditions designed to optimize implicit contributions (i.e., a small distortion size). Participants who trained to reach with the MR distortion exhibited great movement variability, and a subset of participants did not learn to reach with the distortion at all, suggesting that reaching with an MR distortion presents additional challenges compared to reaching with a VR distortion. Successful learning in MR environments may require greater engagement of explicit, strategic processes. Future research is needed to determine which strategies best support learning to reach with an MR distortion and how they are implemented to improve performance.

## Supporting information

S1 Table Summary of each MR-NL participant’s mean angular error (AE; in degrees) in the learning block relative to baseline performance. Each row represents an individual participant’s average AE for early and late trials toward both the left and right targets. Negative values indicate movements in the wrong direction. The final column outlines the rationale for why each participant was designated as an MR-NL participant. Participants needed to have adjusted their reaches to both the left and right targets in the correct direction to be designated as a learner.
(DOCX)

S2 Table Distribution of MR learners (MR-L) and MR non-learners (MR-NL) across classification criteria (N = 20). (DOCX)

S1 Fig Performance during the learning block for the A: MR non-learner group (MR-NL), B: MR learner group (MR-L), C: VR-CW group and D: VR-CCW group. Solid lines represent mean endpoint angular error across trials, with bold lines showing reaches to the right target and thin lines showing reaches to the left target. Shaded regions indicate ±1 standard error of the mean. No-cursor trials within the learning block are overlaid as individual data points. In all panels, no-cursor reaches toward the right target are depicted with circles, and reaches toward the left target are depicted with triangles. In **A** and **B**, for the right target, values approaching −20° are consistent with complete implicit learning, whereas for the left target, values approaching +20° are consistent with complete implicit learning. Negative angular errors reflect reaches to the left side of the target and positive angular errors reflect reaches to the right of the target. In **C** and **D**, for both targets, values approaching +20° are consistent with complete implicit learning.
(TIFF)
