# Supplementary material for: Implicit processes do not contribute to learning to reach in small mirror reversed visuomotor environments
Source: PLoS One. 2026 Jun 8;21(6):e0333564. doi: 10.1371/journal.pone.0333564 (PMC13245788; doi:10.1371/journal.pone.0333564)
Supplement: S1 Table — Each row represents an individual participant’s average AE for early and late trials toward both the left and right targets. Negative values indicate movements in the wrong direction. The final column outlines the rationale for why each participant was designated as an MR-NL participant. Participants needed to have adjusted their reaches to both the left and right targets in the correct direction to be designated as a learner. (DOCX) [file pone.0333564.s001.docx]

|  | Left Target (°) | | Right Target (°) | | Rationale for designation as  MR-NL |
| --- | --- | --- | --- | --- | --- |
| Participant | **Early** | **Late** | **Early** | **Late** |  |
| MR-NL01 | –4.75 | –27.31 | –11.54 | –9.12 | Wrong direction for both targets |
| MR-NL02 | –15.27 | 9.62 | 10.55 | –22.54 | Wrong direction for right target |
| MR-NL03 | 4.74 | 26.04 | –7.86 | –11.07 | Wrong direction for right target |
| MR-NL04 | –16.84 | –25.44 | 20.27 | 6.42 | Wrong direction for left target |
| MR-NL05 | 0.38 | 15.08 | 19.40 | –7.19 | Wrong direction for right target |
| MR-NL06 | –0.13 | 7.03 | 2.44 | –10.85 | Wrong direction for right target |
| MR-NL07 | –11.28 | –14.43 | 22.78 | 0.55 | Right target AEs not different from baseline; wrong direction for left target |
| MR-NL08 | 15.63 | 3.04 | 10.10 | –27.96 | Left target AEs not different from baseline; wrong direction for left target |
| MR-NL09 | –16.99 | 2.90 | 18.71 | 17.49 | Left target AEs not different from baseline |
