# Supplementary material for: Implicit processes do not contribute to learning to reach in small mirror reversed visuomotor environments
Source: PLoS One. 2026 Jun 8;21(6):e0333564. doi: 10.1371/journal.pone.0333564 (PMC13245788; doi:10.1371/journal.pone.0333564)
Supplement: S2 Table — (DOCX) [file pone.0333564.s002.docx]

| Criterion | Number of MR-L participants | Number of MR-NL participants |
| --- | --- | --- |
| 2 SD | 11 | 9 |
| 2.5 SD | 11 | 9 |
| 3 SD | 11 | 9 |
| 4 SD | 8 | 12 |
