# Supplementary figures and images for: Implicit processes do not contribute to learning to reach in small mirror reversed visuomotor environments
Source: PLoS One. 2026 Jun 8;21(6):e0333564. doi: 10.1371/journal.pone.0333564 (PMC13245788; doi:10.1371/journal.pone.0333564)

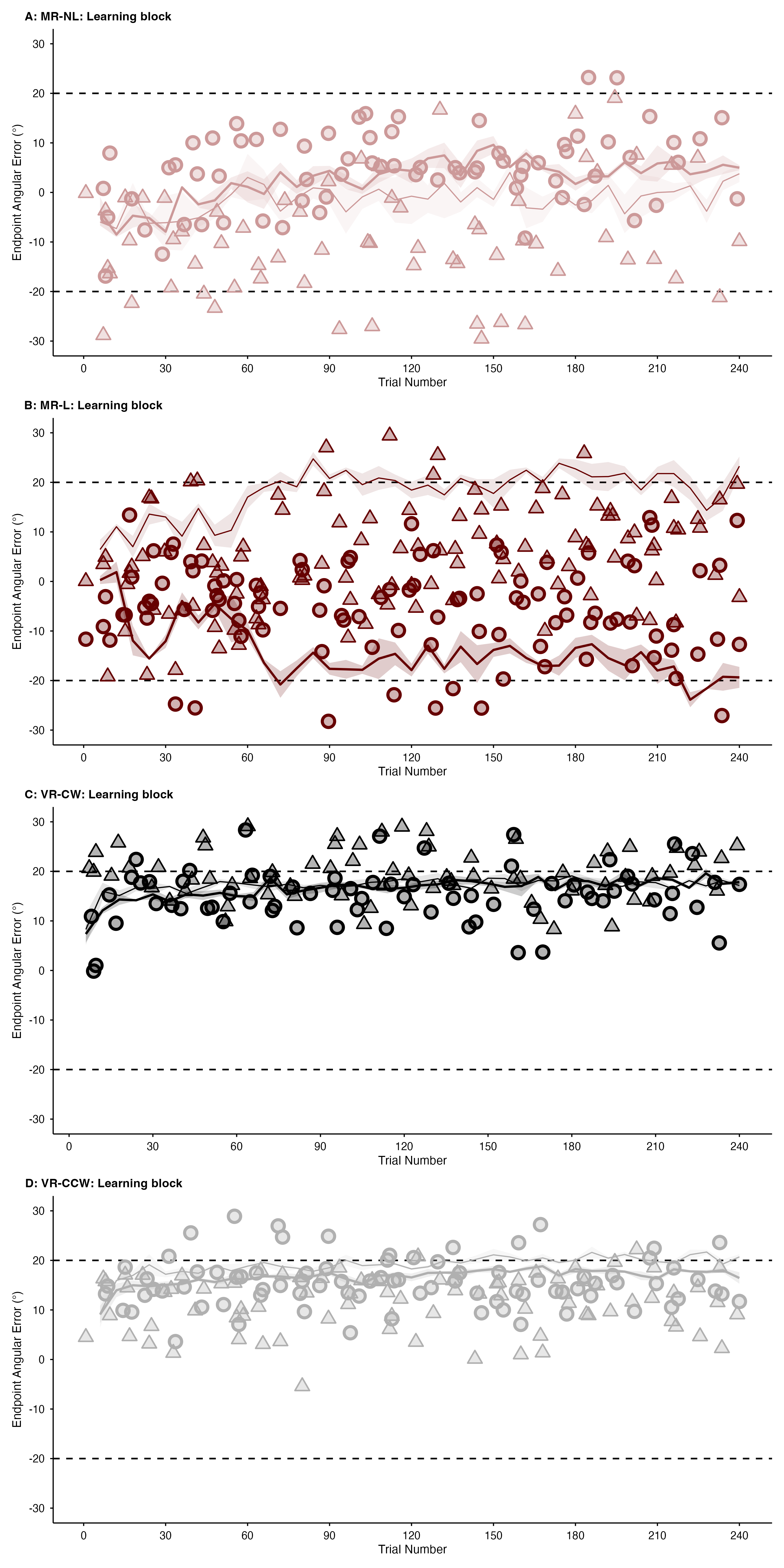

Supplement: S1 Fig — Solid lines represent mean endpoint angular error across trials, with bold lines showing reaches to the right target and thin lines showing reaches to the left target. Shaded regions indicate ±1 standard error of the mean. No-cursor trials within the learning block are overlaid as individual data points. In all panels, no-cursor reaches toward the right target are depicted with circles, and reaches toward the left target are depicted with triangles. In A and B, for the right target, values approaching −20° are consistent with complete implicit learning, whereas for the left target, values approaching +20° are consistent with complete implicit learning. Negative angular errors reflect reaches to the left side of the target and positive angular errors reflect reaches to the right of the target. In C and D, for both targets, values approaching +20° are consistent with complete implicit learning. (TIFF) [file pone.0333564.s003.tiff]
