# Supplementary material for: Implicit processes do not contribute to learning to reach in small mirror reversed visuomotor environments
Source: PLoS One. 2026 Jun 8;21(6):e0333564. doi: 10.1371/journal.pone.0333564 (PMC13245788; doi:10.1371/journal.pone.0333564)
Supplement: Attachment — Submitted filename: Response to Reviewers.pdf [file pone.0333564.s005.pdf]

Response to Reviewers

Manuscript ID: **PONE-D-25-49173**
Title: *Implicit processes do not contribute to learning to reach in small mirror reversed environments*

Dear Dr. Voudouris,

We thank the Academic Editor and the reviewers for their thoughtful and constructive feedback on our manuscript. We have carefully considered all comments and have revised the manuscript accordingly. We believe these revisions have improved the clarity and rigor of the manuscript.

Below we provide a detailed, point-by-point response (format: not bold) to all comments raised by the Academic Editor and the reviewers (format: **bold**). Changes made in the manuscript are indicated in the revised manuscript with tracked changes.

As well, the following statements have been updated:

Data availability statement: All relevant data and analysis code are available at the Open Science Framework (OSF): https://osf.io/wq7p6/. Data have been de-identified in accordance with institutional ethics guidelines.

Funding: This work was supported by Discovery Grants provided by the Natural Sciences and Engineering Research Council of Canada (EKC: RGPIN-2024-03946; GM: RGPIN-2022-03846). The funders had no role in study design, data collection and analysis, decision to publish, or preparation of the manuscript.

Conflict of interest: The authors have declared that no competing interests exist.

Thank you,
Sarvenaz

Responses to Academic Editor and Journal Requirements

1. Please ensure that your manuscript meets PLOS ONE's style requirements, including those for file naming. The PLOS ONE style templates can be found at

https://journals.plos.org/plosone/s/file?id=wjVg/PLOSOne_formatting_sample_main_body.pdf

and

https://journals.plos.org/plosone/s/file?id=ba62/PLOSOne_formatting_sample_title_authors_affiliations.pdf

Thank you for this comment and for providing the formatting guidelines. We have carefully reviewed our manuscript and ensured that it adheres to all PLOS ONE style requirements, including file naming conventions.

2. Please note that PLOS One has specific guidelines on code sharing for submissions in which author-generated code underpins the findings in the manuscript. In these cases, we expect all author-generated code to be made available without restrictions upon publication of the work. Please review our guidelines at https://journals.plos.org/plosone/s/materials-and-software-sharing#loc-sharing-code and ensure that your code is shared in a way that follows best practice and facilitates reproducibility and reuse.

Thank you for this comment. All author-generated code used for the analyses has been made publicly available on the Open Science Framework (OSF). Following the journal guidelines and reviewer feedback, the code has been organized and annotated to improve clarity and usability, thereby facilitating reproducibility and reuse.

Editor comment:

3. Please update your submission to use the PLOS LaTeX template. The template and more information on our requirements for LaTeX submissions can be found at http://journals.plos.org/plosone/s/latex.

Thank you for bringing this to our attention. The manuscript was prepared using Microsoft Word rather than LaTeX. We have carefully reviewed the PLOS ONE formatting guidelines and ensured that the manuscript conforms to the journal's formatting and submission requirements.

4. We note that the grant information you provided in the 'Funding Information' and 'Financial Disclosure' sections do not match.

When you resubmit, please ensure that you provide the correct grant numbers for the awards you received for your study in the 'Funding Information' section.

Thank you for noting this discrepancy. The Funding Information and Financial Disclosure sections have now been revised to ensure consistency. The correct funding statement now reads:

This work was supported by Discovery Grants provided by the Natural Sciences and Engineering Research Council of Canada (EKC: RGPIN-2024-03946; GM: RGPIN-2022-03846). The funders had no role in study design, data collection and analysis, decision to publish, or preparation of the manuscript.

We have added this funding statement to the cover letter.

5. Thank you for stating the following financial disclosure:

"Sarvenaz Heirani Moghaddam received an Ontario graduate scholarship for this work. Drs. Erin Cressman and Gerome Manson's research are funded by NSERC's Discovery Grant program."

Please state what role the funders took in the study. If the funders had no role, please state: "The funders had no role in study design, data collection and analysis, decision to publish, or preparation of the manuscript."

If this statement is not correct you must amend it as needed.

Please include this amended Role of Funder statement in your cover letter; we will change the online submission form on your behalf.

Thank you for this comment. The funding statement has been updated to clarify the role of the funders. Specifically, we now state that the funders had no role in study design, data collection and analysis, decision to publish, or preparation of the manuscript.

6. Please expand the acronym "NSERC" (as indicated in your financial disclosure) so that it states the name of your funders in full.

This information should be included in your cover letter; we will change the online submission form on your behalf.

Thank you for this comment. The acronym NSERC has now been expanded to its full name, Natural Sciences and Engineering Research Council of Canada, in the financial disclosure statement.

7. Thank you for stating the following in your Competing Interests section:

"n/a"

Please complete your Competing Interests on the online submission form to state any Competing Interests. If you have no competing interests, please state "The authors have declared that no competing interests exist.", as detailed online in our guide for authors at http://journals.plos.org/plosone/s/submit-now

This information should be included in your cover letter; we will change the online submission form on your behalf.

Thank you for this comment. The Competing Interests statement has now been updated in the cover letter to read:

The authors have declared that no competing interests exist.

8. We note that Figure 1 in your submission contain copyrighted images. All PLOS content is published under the Creative Commons Attribution License (CC BY 4.0), which means

that the manuscript, images, and Supporting Information files will be freely available online, and any third party is permitted to access, download, copy, distribute, and use these materials in any way, even commercially, with proper attribution. For more information, see our copyright guidelines: http://journals.plos.org/plosone/s/licenses-and-copyright.

We require you to either (1) present written permission from the copyright holder to publish these figures specifically under the CC BY 4.0 license, or (2) remove the figures from your submission:

> **1. You may seek permission from the original copyright holder of Figure 1 to publish the content specifically under the CC BY 4.0 license.**

We recommend that you contact the original copyright holder with the Content Permission Form (http://journals.plos.org/plosone/s/file?id=7c09/content-permission-form.pdf) and the following text:

"I request permission for the open-access journal PLOS ONE to publish XXX under the Creative Commons Attribution License (CCAL) CC BY 4.0 (http://creativecommons.org/licenses/by/4.0/). Please be aware that this license allows unrestricted use and distribution, even commercially, by third parties. Please reply and provide explicit written permission to publish XXX under a CC BY license and complete the attached form."

Please upload the completed Content Permission Form or other proof of granted permissions as an "Other" file with your submission.

In the figure caption of the copyrighted figure, please include the following text: "Reprinted from [ref] under a CC BY license, with permission from [name of publisher], original copyright [original copyright year]."

> **2. If you are unable to obtain permission from the original copyright holder to publish these figures under the CC BY 4.0 license or if the copyright holder's requirements are incompatible with the CC BY 4.0 license, please either i) remove the figure or ii) supply a replacement figure that complies with the CC BY 4.0 license. Please check copyright information on all replacement figures and update the figure caption with source information. If applicable, please specify in the figure caption text when a figure is similar but not identical to the original image and is therefore for illustrative purposes only.**

We have removed the photograph of the Kinarm from Figure 1.

9. Please remove your figures from within your manuscript file, leaving only the individual TIFF/EPS image files, uploaded separately. These will be automatically included in the reviewers' PDF.

Thank you for this note. All figures have now been removed from the manuscript file. The figures have been uploaded separately as individual TIFF files in accordance with the PLOS ONE submission guidelines.

10. Please include captions for your Supporting Information files at the end of your manuscript, and update any in-text citations to match accordingly. Please see our Supporting Information guidelines for more information: http://journals.plos.org/plosone/s/supporting-information.

Thank you for this comment. Captions for all Supporting Information files have been added to the end of the main manuscript (after References). We have also reviewed and updated the in-text citations to ensure they correspond appropriately to the Supporting Information files.

11. We note that there is identifying data in the Supporting Information file < PLOS One Supporting Information.pdf>. Due to the inclusion of these potentially identifying data, we have removed this file from your file inventory. Prior to sharing human research participant data, authors should consult with an ethics committee to ensure data are shared in accordance with participant consent and all applicable local laws.

Data sharing should never compromise participant privacy. It is therefore not appropriate to publicly share personally identifiable data on human research participants. The following are examples of data that should not be shared:

-Name, initials, physical address

-Ages more specific than whole numbers

-Internet protocol (IP) address

-Specific dates (birth dates, death dates, examination dates, etc.)

-Contact information such as phone number or email address

-Location data

-ID numbers that seem specific (long numbers, include initials, titled "Hospital ID") rather than random (small numbers in numerical order)

Data that are not directly identifying may also be inappropriate to share, as in combination they can become identifying. For example, data collected from a small group of participants, vulnerable populations, or private groups should not be shared if they involve indirect identifiers (such as sex, ethnicity, location, etc.) that may risk the identification of study participants.

Additional guidance on preparing raw data for publication can be found in our Data Policy (https://journals.plos.org/plosone/s/data-availability#loc-human-research-participant-data-

and-other-sensitive-data) and in the following article: http://www.bmj.com/content/340/bmj.c181.long.

Please remove or anonymize all personal information (ID numbers that seem specific), ensure that the data shared are in accordance with participant consent, and re-upload a fully anonymized data set. Please note that spreadsheet columns with personal information must be removed and not hidden as all hidden columns will appear in the published file.

Thank you for bringing this to our attention and for removing the file from the submission while the issue was addressed. We have carefully reviewed the Supporting Information dataset and removed all potentially identifying information to ensure participant privacy is protected and that the data comply with PLOS ONE's data-sharing policy for human research participants. Specifically, any variables that could directly or indirectly identify participants have been removed or anonymized. The revised dataset has been checked to ensure that no hidden columns or identifying metadata remain. The fully anonymized dataset has now been re-uploaded as Supporting Information.

12. If the reviewer comments include a recommendation to cite specific previously published works, please review and evaluate these publications to determine whether they are relevant and should be cited. There is no requirement to cite these works unless the editor has indicated otherwise.

Thank you for this note. We carefully evaluated all citation recommendations provided in the reviewer comments and included the suggested references where they were relevant to the present study.

Reviewer #1

Reviewer #1: The manuscript investigates whether implicit processes contribute to learning small mirror reversals compared to small visuomotor rotations. The study addresses a genuine gap in the literature. The finding that successful mirror reversal learners show no implicit aftereffects while rotation learners do appears robust for the specific subset of participants analysed (but see below). The manuscript is overall well written. However, there are fundamental concerns regarding participant exclusion and task comparability that require major revisions to ensure the soundness and validity of the interpretations. Please see below, thank you.

1. Participant exclusion

The most critical issue is the exclusion of 45% of the mirror reversal group (9/20 ppts) as "non-learners", compared to 0% in the VR group. The authors currently relegate this data to supplementary materials, but in reality, the difference in attrition rate is too substantial to be treated as a minor limitation. It fundamentally affects the interpretation of the results and the comparability of the tasks. Excluding nearly half of one experimental group while retaining all participants in the control group is at high risk of creating a selection bias. The

authors should integrate the non-learner data into the main text rather than treating it as supplementary. This includes performing full-sample analyses that incorporate all 20 recruited participants in the mirror reversal group.

Moreover, the 3 SD learning criterion for inclusion appears arbitrary, and thus a sensitivity analysis would be a sensible approach to demonstrate how the results and conclusions change under different inclusion criteria (e.g., 2 SD, 2.5 SD, 4 SD). The goal here is to determine whether the absence of implicit learning is a robust feature of the task or instead an artefact of examining only the most successful learners.

We thank the reviewer for raising this important point. Because the present study was designed to examine the processes supporting successful MR learning, participants were initially classified based on whether they demonstrated learning to reach with the MR distortion or not. That said, we agree with the reviewer that it may appear that our approach introduced selection bias. We have thus now included the MR non-learners as an additional group in our analyses, reporting the results of a 4-group (MR-learners, MR-non-learners, VR-CW, VR-CCW) × 2 target (right, left) × 2 time (early trials, late trials) mixed ANOVA with repeated measures on the last two factors for variables of interest.

In the Data Analyses section, we have modified the following (P. 11, Line 265-276):

"The absolute values of the normalized AEs were compared between groups in a 4 group (MR-learner (MR-L), MR-non-learner (MR-NL), VR-CW, VR-CCW) x 2 target (right, left) x 2 time (early trials, late trials) mixed analysis of variance (ANOVA) with repeated measures (RM) on the last two factors. Participants who trained with VR CW and CCW rotations were considered to form separate groups to preserve target-wise compatibility with MR participants and to maintain comparable sample sizes across groups. We also compared AE variability and mean RT in the learning block across groups in a 4 group (MR-L, MR-NL, VR-CW, VR-CCW) x 2 target (right, left) x 2 time (early trials, late trials) mixed ANOVA with RM on the last two factors. AE variability for each participant was calculated as the standard deviation of AEs across the early or late learning trials for each target. To confirm that participants across all groups complied with the MT criteria, mean MT was analyzed in a 4 group (MR-L, MR-NL, VR-CW, VR-CCW) × 2 target (right, left) × 2 time (early trials, late trials) mixed ANOVA with RM on the last two factors".

In the Results section the following text has been added (P. 13, Line 303-320):

"With respect to learning, ANOVA revealed significant main effects of group ($F(3,38) = 39.245$, $p < 0.001$, $\eta_p^2 = 0.756$) and time ($F(1,38) = 14.913$, $p < 0.001$, $\eta_p^2 = 0.282$), as well as a significant group × time interaction ($F(3,38) = 12.828$, $p < 0.001$, $\eta_p^2 = 0.503$) and group × time × target interaction ($F(3,38) = 5.359$, $p = 0.004$, $\eta_p^2 = 0.297$). Post hoc analysis indicated that early mean AE for the MR-L group (right target: M = 3.48° ± 12.85°, left target: M = 8.56° ± 14.20°) was not different from early mean AE for the MR-NL group (right target: M = 9.04° ± 12.60°, left target: M = -4.95° ± 11.19°) or both the VR-CW (right target: M = 12.28° ± 3.04°, left target: M = 14.94° ± 3.22°), and VR-CCW groups (right target: M = 13.10° ± 2.74°, left target: M = 15.72° ± 2.78°) at both targets (all $p > 0.05$). Mean AE in the late learning trials for the MR-L group (right targe: M = 20.29° ± 3.04°, left target: M = 19.64° ± 7.24°) was significantly greater than their

mean AE in the early learning trials at both targets (both $p < 0.05$), demonstrating changes in reaches. In contrast, the MR-NL group did not show the same increase in mean AE from early to late learning trials (late mean AE for MR-NL: right target: M = -7.14° ± 13.94°, left target: M = -0.39° ± 18.25°; $p = 0.454$). There were no differences in AE between early and late learning trials for either VR group (Late VR-CCW right target: M = 17.98° ± 2.14°, left target: M = 20.16° ± 2.15°; late VR-CW right target: M = 18.50° ± 1.61°, left target: M = 17.76° ± 2.47°; $p > 0.05$), and the magnitude of mean AE in the late learning trials did not differ significantly between the MR-L group and either VR group (both $p > 0.05$; Fig 3A and B)".

These results indicate that the overall pattern of learning remained unchanged when the MR-non-learners were included in the analysis.

"Analysis of AE variability revealed significant main effects of group ($F(3,38) = 56.031$, $p < 0.001$, $\eta_p^2 = 0.816$), target ($F(1,38) = 14.280$, $p < 0.001$, $\eta_p^2 = 0.273$), time ($F(1,38) = 10.449$, $p = 0.003$, $\eta_p^2 = 0.216$), and a significant group x target interaction ($F(3,38) = 4.381$, $p = 0.010$, $\eta_p^2 = 0.257$). Post hoc analysis revealed that the MR-L group (early: M = 14.0° ± 5.2°; late: M = 9.7° ± 4.2°) and the MR-NL group (early: M = 14.3° ± 5.2°; late: M = 10.8° ± 4.0°) demonstrated similar AE variability ($p = 1.000$), while AE variability was significantly greater in both the MR-L and MR-NL groups than the VR-CW (early: M = 5.6° ± 1.1°; late: M = 4.2° ± 1.0°) and the VR-CCW groups (early: M = 4.8° ± 1.1°; late: M = 3.1° ± 0.8°; all $p < 0.001$). Across all groups, AE variability was significantly lower in late learning trials compared to early learning trials ($p < 0.001$). Post hoc analysis of the group x target interaction revealed that AE variability was larger for reaches towards the left target compared to the right target only for the MR-NL group ($p < 0.001$).

Analysis of mean RT in the learning block revealed a significant main effect of group ($F(3,38) = 5.249$, $p = 0.004$, $\eta_p^2 = 0.293$). Post hoc analysis revealed that the MR-L group took longer to initiate their reaches compared to the VR-CW and VR-CCW groups (all $p < 0.05$). No differences were found between the MR-L (early: M = 296.1 ms ± 67.3 ms; late: M = 306.4 ms ± 54.7 ms) and MR-NL groups (early: M = 271.8 ms ± 45.0 ms; late: M = 243.9 ms ± 57.9 ms) or the MR-NL and the VR-CW (early: M = 235.0 ms ± 36.0 ms; late: M = 225.3 ms ± 58.9 ms) and VR-CCW groups (early: M = 238.2 ms ± 31.3 ms; late: M = 218.6 ms ± 53.6 ms; all $p > 0.05$; Fig 3D).

MT data confirmed that all participants adhered to the MT criteria, completing their reaches with MTs ranging from 191 ms to 264 ms throughout the learning block. ANOVA revealed no significant differences between groups ($F(3,38) = 1.555$, $p = 0.216$, $\eta_p^2 = 0.109$). However, there was a significant main effect of target ($F(1,38) = 10.215$, $p = 0.003$, $\eta_p^2 = 0.212$) and a significant group x time interaction ($F(1,38) = 2.917$, $p = 0.047$, $\eta_p^2 = 0.187$). Post hoc analyses revealed that reaches to the right target were approximately 11.6 ms faster than those to the left target but no significant effects associated with the group × time interaction were observed. These results confirm that participants executed their movements in a similar time across groups (MR-L early: M = 238.2 ms ± 46.1 ms; late: M = 198.1 ms ± 24.4 ms; MR-NL early: M = 238.1 ms ± 38.9 ms; late: M = 206.1 ms ± 30.0 ms; VR-CW: early: M = 238.2 ms ± 77.4 ms; late: M = 246.7 ms ± 49.2 ms; VR-CCW: early: M = 242.8 ms ± 44.0 ms; late: M = 257.3 ms ± 53.1 ms) and within the prescribed MT constraints (Fig 3E)."

Figures 3 and 4 have been updated to include data for the MR-NL group.

From the results presented, it is evident that implicit learning was absent among MR participants, regardless of whether they learned to reach with the MR distortion or not. Furthermore, from Figure 3, it is evident that the reaches with cursor feedback differed between our MR-L and MR-NL participants. MR-L participants learned to reach with the cursor distortion, while MR-NL participants did not.

To address the concern that the 3 SD learning criterion was an arbitrary cutoff for classifying participants as learners versus non-learners, we re-classified participants using thresholds of 2, 2.5, 3, and 4 standard deviations. Specifically, participants had to change their reaches to both targets in the correct direction, and the amount of change had to exceed 2, 2.5, 3, and 4 standard deviations relative to baseline reaching errors. Re-classifying participants according to these cut-off criteria led to the following breakdown of the 20 participants into MR-L and MR-NL groups:

Criterion	Number of MR-L participants	Number of MR-NL participants
2 SD	11	9
2.5 SD	11	9
3 SD	11	9
4 SD	8	12

Thus, if we were a bit more liberal with our criteria, we ended up with the same breakdown of participants – the same 11 participants in the MR-L group and 9 participants in the MR-NL group - indicating that the same participants were identified as learners and non-learners across these cut-off criteria. This pattern suggests that participants classified as non-learners did not fall just below the 3 SD threshold; rather, they generally failed to consistently reach in the correct direction for both targets (see Supplementary Table for the rationale for categorizing each participant as an MR non-learner). When the more conservative 4 SD threshold was applied, 3 additional participants were classified as non-learners, for a total of 12 MR-NL participants. Even with this classification, the results remained the same. Specifically, there was an absence of implicit learning across MR-L and MR-NL participants, regardless of the cut-off criteria used. This sensitivity analysis has been added to the Supplementary File.

2. Tasks comparison

The central premise of the study is a comparison between two "matched" 20° distortions. However, there appears to be a fundamental asymmetry in task complexity that challenges this equivalence. A 20° VR provides a time-invariant error signal regardless of reach trajectory. In contrast, a MR represents a dynamic and state-dependent transformation where the error magnitude and direction vary with hand position. The fact that one task yielded a 100% success rate while the other only 55% points strongly to the hypothesis that these tasks may not be matched in difficulty.

The manuscript should explicitly address this asymmetry: the authors need to consider whether the lack of implicit learning in the mirror reversal condition stems from the nature

of the error signal itself, which is a mechanistic explanation distinct from the claim that the task engages a wholly different learning system.

We thank the reviewer for raising an important point regarding the potential asymmetry in the structure of the VR and MR distortions.

We agree that the two distortions differ in the structure of the error signal that they generate. In a VR distortion, the relationship between one's movement direction and visual feedback is rotated by a constant angular offset. In contrast, an MR distortion introduces a state-dependent transformation in which the direction and magnitude of the error signal depend on the hand position relative to body midline and hence can dynamically change over the course of a reach and between reaches. As the reviewer notes, this difference in the structure of the error signal may influence how the motor system responds to the distortion.

It is important to note that the reaching movements in the present study were performed as ballistic "shooting" movements. Participants moved through the target at approximately peak velocity, and visual feedback was removed once the hand crossed the target array. As a result, the time-evolving error signal generated in our MR and VR trials was not expected to influence online movement corrections. Instead, the error signal would be evaluated primarily at the end of the movement and was likely used to inform planning of subsequent reaches.

We agree with the reviewer that differences in the structure of the MR versus VR error signal may have influenced the learning processes engaged. A constant, time-invariant error signal such as that produced by a VR distortion may be well suited for implicit recalibration of an existing sensorimotor mapping. In contrast, the state-dependent error generated by an MR distortion may require participants to determine the appropriate movement direction relative to their body midline, which may encourage the use of explicit strategies. Thus, the absence of implicit learning observed in the MR condition may stem, at least in part, from the structure of the error signal itself and the processes it preferentially recruits, rather than necessarily reflecting engagement of a wholly distinct learning system.

To clarify this issue, we have revised the manuscript to acknowledge that VR and MR distortions differ in the structure of the error signals they generate and that these differences may influence the learning processes engaged. Specifically, we now include the following statement in the Discussion (see P. 18, Line 419-426):

"In a VR task, the discrepancy between expected and actual visual feedback experienced remains constant across the workspace and movement trajectory, providing a stable signal to drive implicit learning. In contrast, an MR distortion produces a state-dependent transformation, in which the magnitude and direction of the error signal depend on the hand position relative to the mirror axis (i.e., body midline). This results in an error signal that changes within and across trials depending on reaching direction relative to the body midline, potentially limiting the ability of a sensory prediction error to drive implicit learning."

3. Statistical power

Given the high exclusion rate as per above, the final sample size of N=11 for the MR group raises concerns about statistical power. On this note, the reported BF10 of 0.340 offers only moderate support for the null hypothesis, not strong. The authors should conduct more robust statistical tests, eg mixed-effects model on the full sample with learning magnitude as covariate, rather than solely relying on ANOVAs of the filtered subset.

We thank the reviewer for raising this important concern regarding statistical power and for suggesting additional analytical approaches.

To address concerns regarding statistical power and the exclusion of MR non-learners, we conducted an additional analysis including the full sample of participants, with learning magnitude (calculated as Late Learning − Early Learning) entered as a covariate.

The linear model revealed a significant main effect of group ($F(3, 37) = 20.53$, $p < 0.001$, $\eta_p^2 = 0.62$), indicating differences in implicit learning across groups. There was no effect of learning magnitude ($F(1, 37) = 0.15$, $p = 0.70$, $\eta_p^2 = 0.004$), and no interaction between group and learning magnitude ($F(3, 34) = 0.42$, $p = 0.74$, $\eta_p^2 = 0.04$). These findings indicate that group differences in implicit learning were not explained by differences in learning magnitude.

Even when including MR non-learners and accounting for variability in learning performance, both MR learners and MR non-learners showed no evidence of implicit adaptation, whereas the VR groups demonstrated robust implicit learning. These results strengthen our original conclusion that implicit processes do not contribute to learning to reach with an MR distortion.

Because these additional analyses yielded conclusions identical to those reported in the manuscript, we have elected not to include them in the main text.

4. RT data

RT is not a pure measure of explicit strategy but could also reflect computational demands, response selection difficulty, motor planning complexity et cetera. The current version of the manuscript implies a direct causal link between elevated reaction times and explicit processing. The authors should revise this language to be more cautious, acknowledging that the data may also reflect the increased cognitive load inherent in calculating the mirror transformation

We thank the reviewer for this helpful comment. We agree that RT should not be interpreted as a pure or direct index of explicit strategy use. As suggested, elevated RTs may also reflect increased computational demands, response selection difficulty, and motor planning complexity.

To address this concern, we have revised the manuscript to use more cautious language when interpreting group differences in RT. Specifically, we modified two sentences in the Discussion that could have been interpreted as implying a direct causal link between RT and explicit processing.

Original sentence #1:

“Previous work has shown that increased RTs are often associated with the engagement of conscious, strategic mechanisms in motor learning”.

Revised sentence (P. 18, Line 432-435):

“Previous research has observed increased RTs during tasks which place greater cognitive or planning demands on the motor system, including situations in which explicit strategies may be engaged during motor learning (28,30–32)”.

Original sentence #2:

“The RT differences, in addition to the lack of implicit learning observed in our study, provide behavioral evidence that MR learning engages distinct processes from VR learning".

Revised sentence (P. 19, Line 442-446):

“The longer RTs observed in our MR-L group, together with the absence of implicit learning, are consistent with the notion that learning to reach with an MR distortion places greater cognitive and planning demands on the motor system comparted to VR learning, and that these demands may involve the engagement of explicit strategies”.

These revisions clarify that elevated RTs may reflect multiple contributing processes and avoid implying a direct causal link between RT and explicit strategy use.

We thank the reviewer for these thoughtful comments, which have improved the clarity and interpretation of the manuscript.

Reviewer #2: This paper explores the nature of adaptation which occurs in response to a small mirror reversal. While small visuomotor rotation perturbations typically elicit implicit learning, it is unknown whether this is the case for a small mirror reversal. Using a Kinarm, small visuomotor rotations or mirror reversals were imposed during target-directed reaching movements. The results demonstrate that while participants in both groups were able to learn the perturbation to the same extent, the mirror reversal group showed virtually no aftereffects. This suggested that mirror reversal learning was not mediated through implicit processes and may occur through different mechanisms than visuomotor rotation learning.

Overall, I find the manuscript to be well-written and interesting. The introduction is clear and sets up the experiment well. The overall conclusions made by the authors are consistent with the findings. I have some questions regarding the methodology and results and suggest some discussion points to consider further.

5. Rotation size: In this paper, a 20-degree rotation is referred to as small. I would argue that many researchers would not consider this a small rotation, as many studies use perturbations as small as a few degrees. Is it referred to as small due to previous literature showing a 20-degree rotation typically only elicits implicit adaptation? This is somewhat dependent on

whether the participant is told about the rotation, and/or given a strategy to use. Please consider clarifying this further in the introduction and defending the notion of 20-degrees being ‘small’.

We thank the reviewer for this helpful comment. We agree that the term “small” can be interpreted differently depending on the context, as visuomotor perturbations used in the literature range from only a few degrees to rotations exceeding 60°. In the present manuscript, we used the term “small” to refer to rotations that primarily engage implicit processes. Prior work has shown that implicit learning processes underlie visuomotor adaptation to cursor rotations of 30° or less, and do not reliably engage explicit strategy use, even when participants are made aware of the cursor distortion and/or are instructed on how to counteract them (e.g., Neville & Cressman, 2018; Werner et al., 2015).

We have revised the Introduction to clarify this point and to better justify our use of the term “small.” Specifically, we added the following to the introduction (P. 4, Line 80-84):

"Implicit processes have been shown to play the dominant role when one learns to reach with a VR distortion of less than 30°, even if one is made aware of the cursor distortion and/or instructed on how to counteract the VR distortion (see 15–17). Given this implicit engagement, and in accordance with previous literature, we refer to distortions of 30° or less as small in magnitude (see 15–17)"

6. Participant instruction: Instructions to the participants during the VR and MR trials have not been reported. Were the participants informed of the perturbation, and if so, what were they instructed to do? Please clarify.

We thank the reviewer for raising this point. Participants were not informed about the presence or the nature of the visuomotor distortion.

We have now clarified this in the Methods section, under VR and MR trials. Specifically, we added the following sentence (P. 9, Line 206-208):

“Participants were not informed about the presence of the visuomotor distortion and were instructed only to move the cursor through the target as quickly and accurately as possible”.

7. Figure 2: In panel B and C, the cursor rotation appears to be less than 20 degrees. Please modify so that the angular difference between the cursor and target is comparable between the VR trials and MR trials. Additionally, you may consider removing the timeline from each individual panel and only keeping it at the very bottom of the figure below E (like a shared x-axis).

We thank the reviewer for this helpful suggestion. We have revised Figure 2 to improve the clarity and accuracy of the visual representation. Specifically, the cursor rotation in panels C and D have been adjusted so that the angular difference between the cursor and the target reflects the 20° rotation used in the current experiment and is comparable to the MR distortion shown in panel B.

In addition, the timeline previously shown in each panel has been removed and is now presented once at the bottom of the figure as a shared timeline for all trial types.

Example of the updated figure:

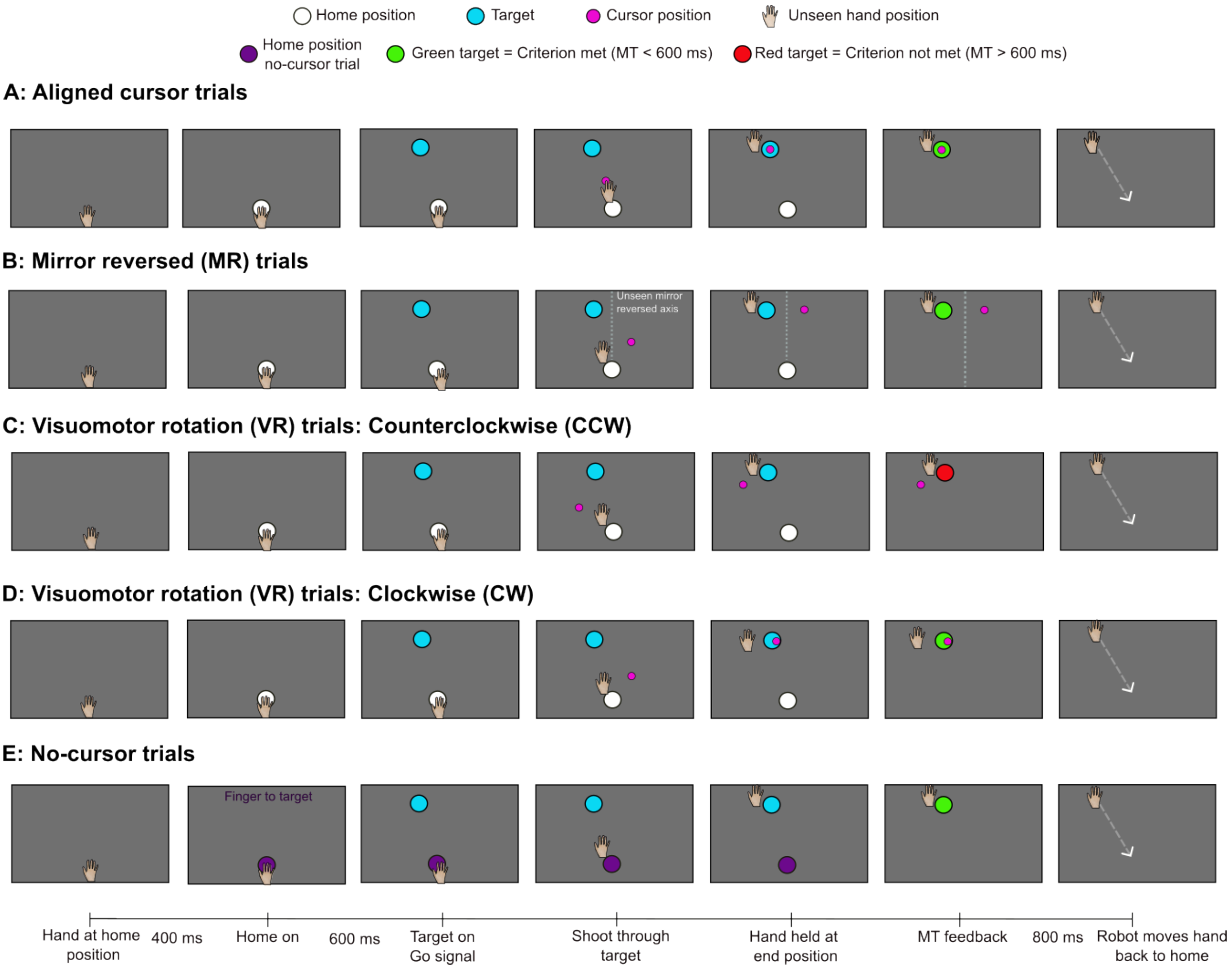

8. Confirmation of learning: What was the justification behind choosing a difference of at least 3 standard deviations between late baseline and late learning as the cut off for determining learning?

Thank you for this comment. Please refer to the response in Comment #1.

9. Experimental groups: In typical VR experiments, adaptation data from CW and CCW groups are collapsed and analyzed together. While I understand that rotation in only one direction is directly comparable to MR for each respective target, please elaborate on why these were analyzed as separate groups, especially considering there was no notable differences between these two rotation directions.

We thank the reviewer for this note. The VR groups were initially analyzed separately because participants experienced rotations in opposite directions (CW vs CCW), and maintaining these

groups enabled us to preserve target-wise compatibility with the MR condition. In addition, keeping the VR-CW and VR-CCW groups separate resulted in more comparable sample sizes across experimental groups.

We have clarified this rationale in the data analysis section (P. 11, Line 268-270):

“Participants who trained with VR CW and CCW rotations were considered to form separate groups to preserve target-wise compatibility with MR participants and to maintain comparable sample sizes across groups”.

10. AE variability: How was AE variability calculated? In any case, only a singular value over 24 trials was obtained for each participant. Considering that the time series of adaptation behaviour is not stationary (follows a curve), this may not be the best method to capture trial-by-trial changes. This is especially important considering the argument made in the discussion that motor variability can suggest exploration and may suggest a mechanism for learning in the MR task. Seeing as this may be an important measure, consider clearly describing how it was calculated, and alternative methods that may be used to fully describe variability behaviour.

We thank the reviewer for this thoughtful comment. AE variability was calculated as the standard deviation of endpoint angular errors across early or late trials within the learning block. Specifically, for each participant we calculated the standard deviation of AE across the early learning trials for each target (12 trials per target) and late learning trials for each target (12 trials per target). These values were then used to compare AE variability across groups and time.

We agree that learning behaviour evolves across trials and that alternative approaches could be used to characterize dynamic changes in variability across the reach training trials (e.g., trial-by-trial analyses). In the present study, however, our goal was to obtain a summary measure of movement variability during the early and late stages of learning to enable direct comparisons across groups. As well, AE variability was included as a secondary measure to characterize behavioural differences between groups, whereas the primary goal of the study was to determine whether implicit processes contributed to learning in the MR task.

To clarify this point, we have revised the Data Analyses section to clearly describe how AE variability was calculated. Specifically, we say (P. 12, Line 272-274):

" AE variability for each participant was calculated as the standard deviation of AEs across the early or late learning trials for each target".

11. Reaction times: Are the reported SD values the SD of mean RT’s between participants or the mean of within-participant RT SD’s? Please specify whether these reflect within or between subject values. Additionally, there seems to be large differences in SD values across groups. Could this be used as another indicator of different strategies being used? Is this something to analyze further?

We thank the reviewer for raising this point. The reported SD values reflect between-participant variability in mean RTs (i.e., the standard deviation of participant mean RTs within each group), rather than within-participant variability across trials. We have clarified this in the Data Analysis section (P. 13, Line 299-300):

“Data are reported as group means and standard deviations, reflecting between participant variability”.

With respect to the variability of RTs across groups, although there are some differences in SD values, these are relatively modest and do not show a consistent pattern across groups or learning (early vs. late). As reported, the MR-L group exhibited longer RTs than the VR groups.

We did compare variability in RT across groups but did not find any significant differences. Thus, we have not included these results in the manuscript and do not interpret these results.

12. Magnitude of AE: mean values for AE magnitude are not reported in the results. Consider including these values when reporting the statistics for learning.

We have now included the mean AE magnitude values in the Results section. Please see Comment #1.

13. Figure 3A: I appreciate that this figure has been separated for each group (keeping CW and CCW separate) and target. However, considering that there were no significant differences between target position or rotation direction, did you consider collapsing across either/both variables for visualization? Considering the main findings from the learning phase revolve around the differences between VR and MR, you may consider adding a visualization where some of these groups are collapsed. Additionally, the SEM’s for each group look tight, even for the MR group. How does this coincide with the increased AE variability in this group?

We thank the reviewer for this helpful suggestion. Following this comment and Comment #1, we have now updated Figure 3A in the Manuscript.

With respect to the relatively small SEM values shown in Figure 3A, these reflect the standard error of the mean endpoint error across participants for each bin of 3 trials during the learning phase. Specifically, SEM was calculated as the SD of the group means for each bin of 3 trials divided by the square root of the number of participants in each group.

In contrast, the AE variability reported for the MR group refers to the standard deviation of the angular error across the first 24 trials (early learning) or last 24 trials (late learning) of the learning block. These SD values were then averaged across participants. Because these measures capture different sources of variability (between-participant SEM vs. within-subject SD), the values are not directly comparable.

14. Implicit adaptation: It appears the exclusion trials within the learning phase were not analyzed. What were these trials intended to tell us? Additionally, there is only 1 figure

showing implicit learning, and it only represents a singular value for each participant. Considering how important implicit adaptation is towards the research question, would there be a way to plot the time course of the exclusion trials over the learning and learning assessment phases?

We thank the reviewer for this thoughtful comment. The no-cursor trials embedded within the learning block were included to probe the extent to which participants showed reach adjustments in the absence of visual feedback while learning trials progressed. In the manuscript, we summarized implicit learning using the mean no-cursor performance from the learning assessment block because this provided a stable post-learning estimate of aftereffects that could be directly compared across groups and targets.

That said, we plotted the time course of no-cursor trials during the learning block. These plots show the evolution of reach behaviour in the absence of visual feedback across the learning block. We have provided the following text and figure in the Supplementary File (P. 3, Line 36-44):

"To further examine learning, we plotted average angular error (AE) of the no-cursor trials across the learning block (see Supplementary Figure 1). These trials assessed reach behaviour in the absence of visual feedback. In the VR groups, no-cursor reaches exhibited a consistent directional bias across trials, consistent with the expression of implicit learning seen in the learning assessment block. In contrast, both MR-L and MR-NL groups showed highly variable reach behaviour, such that there was not a clear distinction in reaches toward the right and left targets in the required direction. This pattern suggests that, in the MR learning block, participants did not develop a stable internal recalibration that could be expressed in the absence of cursor feedback, consistent with the absence of implicit learning observed in the learning assessment block".

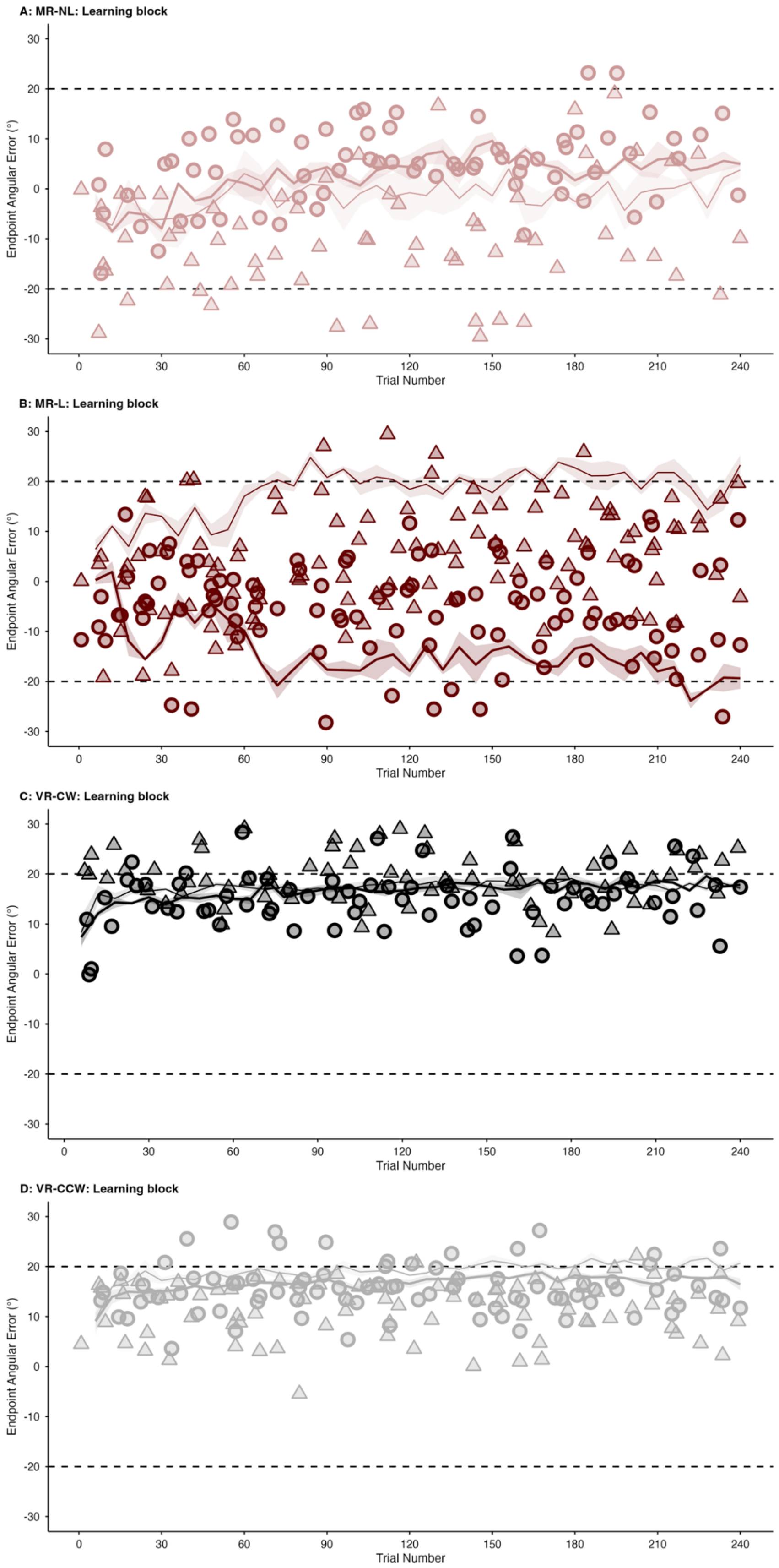
A: MR-NL: Learning block
B: MR-L: Learning block
C: VR-CW: Learning block
D: VR-CCW: Learning block
Endpoint Angular Error (°)
Trial Number
30
20
10
0
-10
-20
-30
0
30
60
90
120
150
180
210
240

Fig S1. Performance during the learning block for the **A**: MR non-learner group (MR-NL), **B**: MR learner group (MR-L), **C**: VR-CW group and **D**: VR-CCW group. Solid lines represent mean endpoint angular error across trials, with bold lines showing reaches to the right target and thin lines showing reaches to the left target. Shaded regions indicate ±1 standard error of the mean. No-cursor trials withing the learning block are overlaid as individual data points. In all panels, no-cursor reaches toward the right target are depicted with circles, and reaches toward the left target are depicted with triangles. In **A** and **B**, for the right target, values approaching -20° are consistent with complete implicit learning, whereas for the left target, values approaching +20° are consistent with complete implicit learning. Negative angular errors reflect reaches to the left side of the target and positive angular errors reflect reaches to the right of the target. In **C** and **D**, for both targets, values approaching +20° are consistent with complete implicit learning.

15. Non-learners: It is quite interesting that nearly half of the participants could not learn the MR perturbation. Given the large number of participants who did not learn, it may be of interest to move some of these data from the supplementary files to the main results (e.g., Figure 1A and 1B). Further, does the claim that "there were no learning related differences between the MR-L and MR-NL groups" refer to the lack of AE variability between groups, or mean AE? Would a lack of difference in AE variability between the groups suggest that although the non-learners were unable to successfully adapt, they were still engaging in similar exploration strategies compared to the learners? Additionally, it appears that many of the 'non-learners' adapted in one target direction. Why might this have been the case? Further speculation/discussion around these issues could enhance these results.

Thank you to the reviewer for this comment. Please see response to Reviewer 1, Comment #1. We have now integrated the MR non-learner (MR-NL) data into the main Results and conducted analyses including all MR participants.

With respect to the statement that "there were no learning-related differences between the MR-L and MR-NL groups," yes, it referred to comparisons of AE variability between the two groups rather than mean AE magnitude in the manuscript. This has now been removed from the supplementary file.

We agree that the behaviour of the MR-NL participants may provide insight into the learning processes engaged during the task. In the revised Discussion, we now acknowledge that although MR-NL participants did not successfully learn to reach with the MR distortion, their movement variability suggests exploratory behaviour during attempts to discover an effective control policy. We also note that some MR-NL participants appeared to learn to reach towards one target, which may reflect the increased complexity of the MR distortion and the possibility of target-specific learning strategies. These points have been added to the Discussion. Specifically, we state the following (P. 19, Line 458-460; P. 20, Line 470-474):

"The increased movement variability observed across MR-L and MR-NL participants in the current study may reflect exploratory behaviour as participants attempted to discover an effective mapping between hand motion and cursor feedback".

"…, several MR-NL participants appeared to adjust their movements to one target in the correct direction. This pattern suggests that the increased complexity of the MR distortion relative to the VR distortion may promote partial or target-specific solutions to emerge before an effective control policy is established".

16. Sensory prediction error (SPE) in the MR task: in the discussion (lines 414-423), it is described that the SPE may be driving implicit adaptation. This raises an interesting question – why is the SPE in the MR task being treated differently? Early work (Mazzoni & Krakauer, 2006) suggested that implicit adaptation to an SPE can override explicit strategy, but perhaps here we are seeing the opposite. Why might this be the case?

We thank the reviewer for raising this interesting point regarding the role of sensory prediction errors (SPEs) in the MR task. Implicit processes are commonly proposed to arise in response to SPEs, defined as the discrepancy between the expected and actual sensory consequences of a movement (Bastian, 2008; Izawa & Shadmehr, 2011). In the present study, SPEs would likely still be generated during MR reaches because the cursor deviates from the expected hand trajectory.

However, an important difference between VR and MR distortions is the structure of the error signal (see Reviewer 1, Comment #2). In a VR task, the cursor deviates from the intended movement direction by a fixed angular offset across target locations, and regardless of where the hand is in the workspace, producing a consistent sensory prediction error that can be gradually corrected through implicit processes, In contrast, in a MR task the relationship between hand motion and visual feedback depends on the hand's position relative to the mirror axis (i.e., body midline), such that both the direction and magnitude of the error vary depending on the location of the hand in the workspace. As a result, although SPEs may be present on each trial, their structure may not provide a stable signal for implicit processes to develop. As suggested, perhaps when reaching with a MR distortion, explicit processes override implicit changes in reaches driven by a SPE.

We have now clarified this point in the Discussion to note that SPEs generated during MR reaches may fail to drive implicit learning despite being present on each trial. Specifically, we state (P. 18, Line 418-426):

"Although sensory prediction errors would likely still arise during MR reaches, the structure of these errors differs from those experienced during VR learning. In a VR task, the discrepancy between expected and actual visual feedback experienced remains constant across the workspace and movement trajectory, providing a stable signal to drive implicit learning. In contrast, an MR distortion produces a state-dependent transformation, in which the magnitude and direction of the error signal depend on the hand position relative to the mirror axis (i.e., body midline). This results in an error signal that changes within and across trials depending on reaching direction relative to the body midline, potentially limiting the ability of a sensory prediction error to drive implicit learning".

17. Number of trials: it can be concluded from the current MR data that no implicit learning occurred, and nearly half the participants were not able to learn it. Could this have anything to do with the number of trials used? Would implicit adaptation potentially develop if there

were more trials or multiple sessions? Additionally, would the non-learners eventually learn the perturbation?

We thank the reviewer for raising this important point. The present study examined learning within a single experimental session. It is possible that additional practice or multiple training sessions could lead to improvements in learning, particularly for participants who did not initially learn to reach with the MR distortion.

Previous work suggests that MR learning may require longer practice compared to VR learning. For example, Telgen et al. (2014) reported that participants required extended practice to learn to reach with an MR compared to a VR distortion. However, even when MR learning occurs with extended training, implicit processes do not appear to play a major role. For example, Wilterson and Taylor (2021) had participants reach with a large MR distortion across five days of practice and found that implicit processes did not meaningfully contribute to reducing errors during MR learning, even after 5 days. Instead, improvements in performance were primarily attributed to the development of explicit strategies.

We now acknowledge this possibility in the Discussion and note that future work could examine whether participants classified as non-learners in the present study would learn the perturbation with extended practice. Specifically, we added (P. 20, Line 474-480):

"It is also possible that the absence of learning in some participants reflects the limited duration of training provided within a single experimental session. Prior work has shown that learning to reach with a large MR distortion may require extended practice compared to learning to reach with a visuomotor rotation (6). However, even with extended training across multiple days, implicit processes do not appear to meaningfully contribute to reducing errors during MR learning (8), suggesting that improvements in MR performance do not rely on implicit processes even after 5 days of reaches" (P. 20, Line 474-480).

Additional comments:

1. Line 58: bracket missing
2. Line 126: delete 'were'
3. Line 399: typo: 'contributed'
4. Typo in supplementary figure 1A: "MR-NL left target" label; "L" not capitalized
5. Ensure citation style stays consistent throughout the paper (Vancouver vs APA)
6. I would suggest adding a README file to your OSF page. Although all the data is accessible, it is not clear what values are given in each excel sheet. Additionally, I would suggest ensuring the code will run without edits from the user (e.g., they will have a different working directory). You may also consider editing the comments in the code to tell the reader which figure is being generated (e.g., "Figure 3"). These changes will help make your data and code more accessible.

We thank the reviewer for these helpful comments and suggestions. The manuscript and associated materials have been revised accordingly.

1. The missing bracket on line 58 has been corrected.
2. The extra word “were” on line 126 has been removed.
3. The typo on line 399 has been corrected.
4. This figure has now been removed from the supplementary file.
5. We reviewed the manuscript to ensure that the citation style is consistent throughout.
6. In response to the reviewer’s suggestion, we added a README file to the OSF repository describing the contents of each Excel sheet and clarifying which data are used for each figure. We also revised the analysis script so that it no longer depends on a user-specific working directory and added section headers/comments indicating which figure is generated by each block of code. These changes improve the accessibility and reproducibility of the shared data and analysis pipeline.
