# Supplementary material for: Implicit processes do not contribute to learning to reach in small mirror reversed visuomotor environments
Source: PLoS One. 2026 Jun 8;21(6):e0333564. doi: 10.1371/journal.pone.0333564 (PMC13245788; doi:10.1371/journal.pone.0333564)
Supplement: Attachment — Submitted filename: Response to Reviewers.docx [file pone.0333564.s006.docx]

**Response to Reviewers**

Dear Dr. Voudouris,

Thank you for the positive feedback regarding our manuscript (**PONE-D-25-49173R1**).

Below, we provide a detailed point-by-point response to all comments raised by the Academic Editor and reviewers. Our responses are presented in regular text, while reviewer comments are shown in **bold**. All changes made in the revised manuscript are indicated using tracked changes.

Thank you for your time and consideration.

Sincerely,

Sarvenaz Heirani Moghaddam

**Journal Requirements:**

**1. If the reviewer comments include a recommendation to cite specific previously published works, please review and evaluate these publications to determine whether they are relevant and should be cited. There is no requirement to cite these works unless the editor has indicated otherwise.** 

Thank you to the Editor for this note. We now site Lee & Wagenmakers, 2013 when reporting our Bayesian analysis results.

**2. Please review your reference list to ensure that it is complete and correct. If you have cited papers that have been retracted, please include the rationale for doing so in the manuscript text, or remove these references and replace them with relevant current references. Any changes to the reference list should be mentioned in the rebuttal letter that accompanies your revised manuscript. If you need to cite a retracted article, indicate the article’s retracted status in the References list and also include a citation and full reference for the retraction notice.**

We have updated our reference list to include all in-text citations. We have not cited any retracted articles.

**Review Comments to the Author**

**Reviewer #1: I thank the authors for their thorough revision. I am happy to accept, with just one final comment: the Bayes factors for the MR-L and MR-NL groups (BF₁₀ = 0.340 and BF₁₀ = 0.324, respectively) really correspond to anecdotal evidence for the null on standard scales (e.g., Lee & Wagenmakers, 2013), that is, just below the conventional threshold for moderate evidence. The authors may wish to relate the BF values to a standard evidence scale when describing the strength of support for the null**

Thank you to the Reviewer for this comment. We have updated the reporting of our Bayesian analysis to indicate “anecdotal-to-moderate” evidence in favor of the null hypothesis, in accordance wtih Lee & Wagenmakers, 2014. Specifically, we now state (P. 16, Line 381):

“Bayesian one sample *t*-tests indicated anecdotal-to-moderate support for the null hypothesis (23) in both the MR-L group and the MR-NL group (all *p* > 0.05; MR-L: *BF₁₀* = 0.340; MR-NL: *BF₁₀* = 0.324), suggesting that implicit learning was not significantly different from zero in either the MR-L or the MR-NL groups.”

**Reviewer #2: I would like to thank the authors for their thorough integration of my previous comments. I only have minor comments remaining.

AE Variability calculation: While I thank the authors for clarifying how they calculated angular variability, I still have some questions regarding this measure. Calculating variability by taking the SD over 24 trials may be a reasonable method for late learning, as participants have reached a plateau. However, during early learning, the SD doesn't necessarily tell us about the variability in reaching movements, since the mean is changing (as they are learning to adapt). This is further exemplified when stating that there was greater variability in early learning compared to late. This could simply be due to the fact that the AE values during early learning are much more spread out as the participant adapts. Is this a limitation in the variability measure? If so, consider identifying this limitation within the manuscript. Alternatively, other measures could be used to determine variability, such as performing a simple curve fit and using the mean residuals based on the fit as a measure of variability.**

Thank you to the Reviewer for this comment. As indicated, early adaptation would be associated with greater variability as there is great adjustment in participants’ movements as they look to compensate for the visuomotor distortion. We have now noted this limitation in our discussion. Furthermore, we indicate that variability continues to be higher for our MR participants compared to our VR participants even in late learning trials, suggesting that participants did not achieve a plateau and continued to adjust their movements. We have now added the following to our discussion (P. 19, Line 466):

“The increased movement variability observed in MR participants compared to VR participants may be a signature of the motor system’s attempt to explore and establish a new sensorimotor mapping under conditions where implicit learning mechanisms are not engaged. This increased variability was observed across both early and late learning trials (i.e., the standard deviation of the first 24 (early) and last 24 (late) learning trials), even though mean AE did not differ significantly between the MR-L and VR groups. We interpret our variability estimates during early learning cautiously, as participants were learning to reach with the MR or VR distortion and AE would be expected to change across trials. However, the sustained increase in variability even in late learning trials suggests performance by MR participants did not stabilize to the same extent as VR participants, and participants continued to adjust their reaches from trial to trial.”

**Figure 3A: It appears that the VR CW group is missing from figure 3A. Please amend this figure or clarify if any groups have been collapsed here in the figure caption.**

Thank you to the Reviewer for this comment. We have updated Figure 3A to include the VR CW group.
