# Supplementary material for: Implicit processes do not contribute to learning to reach in small mirror reversed visuomotor environments
Source: PLoS One. 2026 Jun 8;21(6):e0333564. doi: 10.1371/journal.pone.0333564 (PMC13245788; doi:10.1371/journal.pone.0333564)
Supplement: Peer review history

# Peer review: Decision Letter 0

**DOI:** 10.1371/journal.pone.0333564.r001

**Authors:** Dimitris Voudouris

19 Dec 2025

PONE-D-25-49173

Implicit processes do not contribute to learning to reach in small mirror reversed environments

PLOS One

Dear Dr. Heirani Moghaddam,

Thank you for submitting your manuscript to PLOS ONE. I have received the reports from our two reviewers, and you can see their detailed comments below. They both find the study interesting and relevant, but they raise important points that you are invited to address by revising the manuscript. These mainly refer to revisions on the methods and analyses that can strengthen the manuscript. Please see more details below.

Please submit your revised manuscript by Feb 02 2026 11:59PM. If you will need more time than this to complete your revisions, please reply to this message or contact the journal office at plosone@plos.org. When you're ready to submit your revision, log on to https://www.editorialmanager.com/pone/ and select the 'Submissions Needing Revision' folder to locate your manuscript file.

Please include the following items when submitting your revised manuscript:

- A letter that responds to each point raised by the academic editor and reviewer(s). You should upload this letter as a separate file labeled 'Response to Reviewers'.
- A marked-up copy of your manuscript that highlights changes made to the original version. You should upload this as a separate file labeled 'Revised Manuscript with Track Changes'.
- An unmarked version of your revised paper without tracked changes. You should upload this as a separate file labeled 'Manuscript'.

If you would like to make changes to your financial disclosure, please include your updated statement in your cover letter. Guidelines for resubmitting your figure files are available below the reviewer comments at the end of this letter.

If applicable, we recommend that you deposit your laboratory protocols in protocols.io to enhance the reproducibility of your results. Protocols.io assigns your protocol its own identifier (DOI) so that it can be cited independently in the future. For instructions see: https://journals.plos.org/plosone/s/submission-guidelines#loc-laboratory-protocols. Additionally, PLOS ONE offers an option for publishing peer-reviewed Lab Protocol articles, which describe protocols hosted on protocols.io. Read more information on sharing protocols at https://plos.org/protocols?utm_medium=editorial-email&utm_source=authorletters&utm_campaign=protocols.

We look forward to receiving your revised manuscript.

Kind regards,

Dimitris Voudouris

Academic Editor

PLOS One

**Journal Requirements:**

When submitting your revision, we need you to address these additional requirements.

1. Please ensure that your manuscript meets PLOS ONE's style requirements, including those for file naming. The PLOS ONE style templates can be found at

https://journals.plos.org/plosone/s/file?id=wjVg/PLOSOne_formatting_sample_main_body.pdf and

https://journals.plos.org/plosone/s/file?id=ba62/PLOSOne_formatting_sample_title_authors_affiliations.pdf

2. Please note that PLOS One has specific guidelines on code sharing for submissions in which author-generated code underpins the findings in the manuscript. In these cases, we expect all author-generated code to be made available without restrictions upon publication of the work. Please review our guidelines at https://journals.plos.org/plosone/s/materials-and-software-sharing#loc-sharing-code and ensure that your code is shared in a way that follows best practice and facilitates reproducibility and reuse.

3. Please update your submission to use the PLOS LaTeX template. The template and more information on our requirements for LaTeX submissions can be found at http://journals.plos.org/plosone/s/latex.

4. We note that the grant information you provided in the ‘Funding Information’ and ‘Financial Disclosure’ sections do not match.

When you resubmit, please ensure that you provide the correct grant numbers for the awards you received for your study in the ‘Funding Information’ section.

5. Thank you for stating the following financial disclosure:

“Sarvenaz Heirani Moghaddam received Ontario graduate scholarship for this work. Drs. Erin Cressman and Gerome Manson's laboratories are funded by the Discovery Grant (NSERC).”

Please state what role the funders took in the study.  If the funders had no role, please state: "The funders had no role in study design, data collection and analysis, decision to publish, or preparation of the manuscript."

If this statement is not correct you must amend it as needed.

Please include this amended Role of Funder statement in your cover letter; we will change the online submission form on your behalf.

6. Please expand the acronym “NSERC” (as indicated in your financial disclosure) so that it states the name of your funders in full.

This information should be included in your cover letter; we will change the online submission form on your behalf.

7. Thank you for stating the following in your Competing Interests section:

“n/a”

Please complete your Competing Interests on the online submission form to state any Competing Interests. If you have no competing interests, please state "The authors have declared that no competing interests exist.", as detailed online in our guide for authors at http://journals.plos.org/plosone/s/submit-now

This information should be included in your cover letter; we will change the online submission form on your behalf.

8. We note that Figure 1 in your submission contain copyrighted images. All PLOS content is published under the Creative Commons Attribution License (CC BY 4.0), which means that the manuscript, images, and Supporting Information files will be freely available online, and any third party is permitted to access, download, copy, distribute, and use these materials in any way, even commercially, with proper attribution. For more information, see our copyright guidelines: http://journals.plos.org/plosone/s/licenses-and-copyright.

We require you to either (1) present written permission from the copyright holder to publish these figures specifically under the CC BY 4.0 license, or (2) remove the figures from your submission:

1. You may seek permission from the original copyright holder of Figure 1 to publish the content specifically under the CC BY 4.0 license.

We recommend that you contact the original copyright holder with the Content Permission Form (http://journals.plos.org/plosone/s/file?id=7c09/content-permission-form.pdf) and the following text:

“I request permission for the open-access journal PLOS ONE to publish XXX under the Creative Commons Attribution License (CCAL) CC BY 4.0 (http://creativecommons.org/licenses/by/4.0/). Please be aware that this license allows unrestricted use and distribution, even commercially, by third parties. Please reply and provide explicit written permission to publish XXX under a CC BY license and complete the attached form.”

Please upload the completed Content Permission Form or other proof of granted permissions as an "Other" file with your submission.

In the figure caption of the copyrighted figure, please include the following text: “Reprinted from [ref] under a CC BY license, with permission from [name of publisher], original copyright [original copyright year].”

2. If you are unable to obtain permission from the original copyright holder to publish these figures under the CC BY 4.0 license or if the copyright holder’s requirements are incompatible with the CC BY 4.0 license, please either i) remove the figure or ii) supply a replacement figure that complies with the CC BY 4.0 license. Please check copyright information on all replacement figures and update the figure caption with source information. If applicable, please specify in the figure caption text when a figure is similar but not identical to the original image and is therefore for illustrative purposes only.

9. Please remove your figures from within your manuscript file, leaving only the individual TIFF/EPS image files, uploaded separately. These will be automatically included in the reviewers’ PDF.

10. Please include captions for your Supporting Information files at the end of your manuscript, and update any in-text citations to match accordingly. Please see our Supporting Information guidelines for more information: http://journals.plos.org/plosone/s/supporting-information.

11. We note that there is identifying data in the Supporting Information file < PLOS One Supporting Information.pdf>. Due to the inclusion of these potentially identifying data, we have removed this file from your file inventory. Prior to sharing human research participant data, authors should consult with an ethics committee to ensure data are shared in accordance with participant consent and all applicable local laws.

Data sharing should never compromise participant privacy. It is therefore not appropriate to publicly share personally identifiable data on human research participants. The following are examples of data that should not be shared:

-Name, initials, physical address

-Ages more specific than whole numbers

-Internet protocol (IP) address

-Specific dates (birth dates, death dates, examination dates, etc.)

-Contact information such as phone number or email address

-Location data

-ID numbers that seem specific (long numbers, include initials, titled “Hospital ID”) rather than random (small numbers in numerical order)

Data that are not directly identifying may also be inappropriate to share, as in combination they can become identifying. For example, data collected from a small group of participants, vulnerable populations, or private groups should not be shared if they involve indirect identifiers (such as sex, ethnicity, location, etc.) that may risk the identification of study participants.

Additional guidance on preparing raw data for publication can be found in our Data Policy (https://journals.plos.org/plosone/s/data-availability#loc-human-research-participant-data-and-other-sensitive-data) and in the following article: http://www.bmj.com/content/340/bmj.c181.long.

Please remove or anonymize all personal information (ID numbers that seem specific), ensure that the data shared are in accordance with participant consent, and re-upload a fully anonymized data set. Please note that spreadsheet columns with personal information must be removed and not hidden as all hidden columns will appear in the published file.

12. If the reviewer comments include a recommendation to cite specific previously published works, please review and evaluate these publications to determine whether they are relevant and should be cited. There is no requirement to cite these works unless the editor has indicated otherwise.

[Note: HTML markup is below. Please do not edit.]

Reviewers' comments:

Reviewer's Responses to Questions

**Comments to the Author**

1. Is the manuscript technically sound, and do the data support the conclusions?

The manuscript must describe a technically sound piece of scientific research with data that supports the conclusions. Experiments must have been conducted rigorously, with appropriate controls, replication, and sample sizes. The conclusions must be drawn appropriately based on the data presented.

Reviewer #1: Partly

Reviewer #2: Yes

**********

2. Has the statistical analysis been performed appropriately and rigorously?

Reviewer #1: No

Reviewer #2: Yes

**********

3. Have the authors made all data underlying the findings in their manuscript fully available?

The PLOS Data policy requires authors to make all data underlying the findings described in their manuscript fully available without restriction, with rare exception (please refer to the Data Availability Statement in the manuscript PDF file). The data should be provided as part of the manuscript or its supporting information, or deposited to a public repository. For example, in addition to summary statistics, the data points behind means, medians and variance measures should be available. If there are restrictions on publicly sharing data—e.g. participant privacy or use of data from a third party—those must be specified.

Reviewer #1: Yes

Reviewer #2: Yes

**********

4. Is the manuscript presented in an intelligible fashion and written in standard English?

PLOS ONE does not copyedit accepted manuscripts, so the language in submitted articles must be clear, correct, and unambiguous. Any typographical or grammatical errors should be corrected at revision, so please note any specific errors here.

Reviewer #1: Yes

Reviewer #2: Yes

**********

5. Review Comments to the Author

Please use the space provided to explain your answers to the questions above. You may also include additional comments for the author, including concerns about dual publication, research ethics, or publication ethics. (Please upload your review as an attachment if it exceeds 20,000 characters)

Reviewer #1: The manuscript investigates whether implicit processes contribute to learning small mirror reversals compared to small visuomotor rotations. The study addresses a genuine gap in the literature. The finding that successful mirror reversal learners show no implicit aftereffects while rotation learners do appears robust for the specific subset of participants analysed (but see below). The manuscript is overall well written. However, there are fundamental concerns regarding participant exclusion and task comparability that require major revisions to ensure the soundness and validity of the interpretations. Please see below, thank you.

1. Participant exclusion

The most critical issue is the exclusion of 45% of the mirror reversal group (9/20 ppts) as "non-learners", compared to 0% in the VR group. The authors currently relegate this data to supplementary materials, but in reality the difference in attrition rate is too substantial to be treated as a minor limitation. It fundamentally affects the interpretation of the results and the comparability of the tasks. Excluding nearly half of one experimental group while retaining all participants in the control group is at high risk of creating a selection bias. The authors should integrate the non-learner data into the main text rather than treating it as supplementary. This includes performing full-sample analyses that incorporate all 20 recruited participants in the mirror reversal group.

Moreover, the 3 SD learning criterion for inclusion appears arbitrary and thus a sensitivity analysis would be a sensible approach to demonstrate how the results and conclusions change under different inclusion criteria (eg 2 SD, 2.5 SD, 4 SD). The goal here is to determine whether the absence of implicit learning is a robust feature of the task or instaed an artefact of examining only the most successful learners.

2. Tasks comparison

The central premise of the study is a comparison between two "matched" 20° distortions. However, there appears to be a fundamental asymmetry in task complexity that challenges this equivalence. A 20° VR provides a time-invariant error signal regardless of reach trajectory. In contrast, a MR represents a dynamic and state-dependent transformation where the error magnitude and direction vary with hand position. The fact that one task yielded a 100% success rate while the other only 55% points strongly to the hypothesis that these tasks may not be matched in difficulty.

The manuscript should explicitly address this asymmetry: the authors need to consider whether the lack of implicit learning in the mirror reversal condition stems from the nature of the error signal itself, which is a mechanistic explanation distinct from the claim that the task engages a wholly different learning system.

3. Statistical power

Given the high exclusion rate as per above, the final sample size of N=11 for the MR group raises concerns about statistical power. On this note, the reported BF10 of 0.340 offers only moderate support for the null hypothesis, not strong. The authors should conduct more robust statistical tests, eg mixed-effects model on the full sample with learning magnitude as covariate, rather than solely relying on ANOVAs of the filtered subset.

4. RT data

RT is not a pure measure of explicit strategy but could also reflect computational demands, response selection difficulty, motor planning complexity et cetera. The current version of the manuscript implies a direct causal link between elevated reaction times and explicit processing. The authors should revise this language to be more cautious, acknowledging that the data may also reflect the increased cognitive load inherent in calculating the mirror transformation

Reviewer #2: This paper explores the nature of adaptation which occurs in response to a small mirror reversal. While small visuomotor rotation perturbations typically elicit implicit learning, it is unknown whether this is the case for a small mirror reversal. Using a Kinarm, small visuomotor rotations or mirror reversals were imposed during target-directed reaching movements. The results demonstrate that while participants in both groups were able to learn the perturbation to the same extent, the mirror reversal group showed virtually no aftereffects. This suggested that mirror reversal learning was not mediated through implicit processes and may occur through different mechanisms than visuomotor rotation learning.

Overall, I find the manuscript to be well-written and interesting. The introduction is clear and sets up the experiment well. The overall conclusions made by the authors are consistent with the findings. I have some questions regarding the methodology and results and suggest some discussion points to consider further.

Rotation size: In this paper, a 20-degree rotation is referred to as small. I would argue that many researchers would not consider this a small rotation, as many studies use perturbations as small as a few degrees. Is it referred to as small due to previous literature showing a 20-degree rotation typically only elicits implicit adaptation? This is somewhat dependent on whether the participant is told about the rotation, and/or given a strategy to use. Please consider clarifying this further in the introduction and defending the notion of 20-degrees being ‘small’.

Participant instruction: Instructions to the participants during the VR and MR trials have not been reported. Were the participants informed of the perturbation, and if so, what were they instructed to do? Please clarify.

Figure 2: In panel B and C, the cursor rotation appears to be less than 20 degrees. Please modify so that the angular difference between the cursor and target is comparable between the VR trials and MR trials. Additionally, you may consider removing the timeline from each individual panel and only keeping it at the very bottom of the figure below E (like a shared x-axis).

Confirmation of learning: What was the justification behind choosing a difference of at least 3 standard deviations between late baseline and late learning as the cut off for determining learning?

Experimental groups: In typical VR experiments, adaptation data from CW and CCW groups are collapsed and analyzed together. While I understand that rotation in only one direction is directly comparable to MR for each respective target, please elaborate on why these were analyzed as separate groups, especially considering there was no notable differences between these two rotation directions.

AE variability: How was AE variability calculated? In any case, only a singular value over 24 trials was obtained for each participant. Considering that the time series of adaptation behaviour is not stationary (follows a curve), this may not be the best method to capture trial-by-trial changes. This is especially important considering the argument made in the discussion that motor variability can suggest exploration and may suggest a mechanism for learning in the MR task. Seeing as this may be an important measure, consider clearly describing how it was calculated, and alternative methods that may be used to fully describe variability behaviour.

Reaction times: Are the reported SD values the SD of mean RT’s between participants or the mean of within-participant RT SD’s? Please specify whether these reflect within or between subject values. Additionally, there seems to be large differences in SD values across groups. Could this be used as another indicator of different strategies being used? Is this something to analyze further?

Magnitude of AE: mean values for AE magnitude are not reported in the results. Consider including these values when reporting the statistics for learning.

Figure 3A: I appreciate that this figure has been separated for each group (keeping CW and CCW separate) and target. However, considering that there were no significant differences between target position or rotation direction, did you consider collapsing across either/both variables for visualization? Considering the main findings from the learning phase revolve around the differences between VR and MR, you may consider adding a visualization where some of these groups are collapsed. Additionally, the SEM’s for each group look tight, even for the MR group. How does this coincide with the increased AE variability in this group?

Implicit adaptation: It appears the exclusion trials within the learning phase were not analyzed. What were these trials intended to tell us? Additionally, there is only 1 figure showing implicit learning, and it only represents a singular value for each participant. Considering how important implicit adaptation is towards the research question, would there be a way to plot the time course of the exclusion trials over the learning and learning assessment phases?

Non-learners: It is quite interesting that nearly half of the participants could not learn the MR perturbation. Given the large number of participants who did not learn, it may be of interest to move some of these data from the supplementary files to the main results (e.g., Figure 1A and 1B). Further, does the claim that “there were no learning related differences between the MR-L and MR-NL groups” refer to the lack of AE variability between groups, or mean AE? Would a lack of difference in AE variability between the groups suggest that although the non-learners were unable to successfully adapt, they were still engaging in similar exploration strategies compared to the learners? Additionally, it appears that many of the ‘non-learners’ adapted in one target direction. Why might this have been the case? Further speculation/discussion around these issues could enhance these results.

Sensory prediction error (SPE) in the MR task: in the discussion (lines 414-423), it is described that the SPE may be driving implicit adaptation. This raises an interesting question – why is the SPE in the MR task being treated differently? Early work (Mazzoni & Krakauer, 2006) suggested that implicit adaptation to an SPE can override explicit strategy, but perhaps here we are seeing the opposite. Why might this be the case?

Number of trials: it can be concluded from the current MR data that no implicit learning occurred, and nearly half the participants were not able to learn it. Could this have anything to do with the number of trials used? Would implicit adaptation potentially develop if there were more trials or multiple sessions? Additionally, would the non-learners eventually learn the perturbation?

Additional comments:

1. Line 58: bracket missing

2. Line 126: delete ‘were’

3. Line 399: typo: ‘contributed’

4. Typo in supplementary figure 1A: “MR-NL left target” label; “L” not capitalized

5. Ensure citation style stays consistent throughout the paper (Vancouver vs APA)

6. I would suggest adding a README file to your OSF page. Although all the data is accessible, it is not clear what values are given in each excel sheet. Additionally, I would suggest ensuring the code will run without edits from the user (e.g., they will have a different working directory). You may also consider editing the comments in the code to tell the reader which figure is being generated (e.g., “Figure 3”). These changes will help make your data and code more accessible.

**********

6. PLOS authors have the option to publish the peer review history of their article (what does this mean?). If published, this will include your full peer review and any attached files.

If you choose “no”, your identity will remain anonymous but your review may still be made public.

**Do you want your identity to be public for this peer review?** For information about this choice, including consent withdrawal, please see our Privacy Policy.

Reviewer #1: No

Reviewer #2: No

**********

[NOTE: If reviewer comments were submitted as an attachment file, they will be attached to this email and accessible via the submission site. Please log into your account, locate the manuscript record, and check for the action link "View Attachments". If this link does not appear, there are no attachment files.]

To ensure your figures meet our technical requirements, please review our figure guidelines: https://journals.plos.org/plosone/s/figures

You may also use PLOS’s free figure tool, NAAS, to help you prepare publication quality figures: https://journals.plos.org/plosone/s/figures#loc-tools-for-figure-preparation.

NAAS will assess whether your figures meet our technical requirements by comparing each figure against our figure specifications.

# Author comment: Author response to Decision Letter 1

**DOI:** 10.1371/journal.pone.0333564.r002

31 Mar 2026

Response to Reviewers

Manuscript ID: PONE-D-25-49173

Title: Implicit processes do not contribute to learning to reach in small mirror reversed environments

Dear Dr. Voudouris,

We thank the Academic Editor and the reviewers for their thoughtful and constructive feedback on our manuscript. We have carefully considered all comments and have revised the manuscript accordingly. We believe these revisions have improved the clarity and rigor of the manuscript.

Below we provide a detailed, point-by-point response (format: not bold) to all comments raised by the Academic Editor and the reviewers (format: bold). Changes made in the manuscript are indicated in the revised manuscript with tracked changes.

As well, the following statements have been updated:

Data availability statement: All relevant data and analysis code are available at the Open Science Framework (OSF): https://osf.io/wq7p6/. Data have been de-identified in accordance with institutional ethics guidelines.

Funding: This work was supported by Discovery Grants provided by the Natural Sciences and Engineering Research Council of Canada (EKC: RGPIN-2024-03946; GM: RGPIN-2022-03846). The funders had no role in study design, data collection and analysis, decision to publish, or preparation of the manuscript.

Conflict of interest: The authors have declared that no competing interests exist.

Thank you,

Sarvenaz

Responses to Academic Editor and Journal Requirements

1. Please ensure that your manuscript meets PLOS ONE's style requirements, including those for file naming. The PLOS ONE style templates can be found at

https://journals.plos.org/plosone/s/file?id=wjVg/PLOSOne_formatting_sample_main_body.pdf

and

https://journals.plos.org/plosone/s/file?id=ba62/PLOSOne_formatting_sample_title_authors_affiliations.pdf

Thank you for this comment and for providing the formatting guidelines. We have carefully reviewed our manuscript and ensured that it adheres to all PLOS ONE style requirements, including file naming conventions.

2. Please note that PLOS One has specific guidelines on code sharing for submissions in which author-generated code underpins the findings in the manuscript. In these cases, we expect all author-generated code to be made available without restrictions upon publication of the work. Please review our guidelines at https://journals.plos.org/plosone/s/materials-and-software-sharing#loc-sharing-code and ensure that your code is shared in a way that follows best practice and facilitates reproducibility and reuse.

Thank you for this comment. All author-generated code used for the analyses has been made publicly available on the Open Science Framework (OSF). Following the journal guidelines and reviewer feedback, the code has been organized and annotated to improve clarity and usability, thereby facilitating reproducibility and reuse.

Editor comment:

3. Please update your submission to use the PLOS LaTeX template. The template and more information on our requirements for LaTeX submissions can be found at http://journals.plos.org/plosone/s/latex.

Thank you for bringing this to our attention. The manuscript was prepared using Microsoft Word rather than LaTeX. We have carefully reviewed the PLOS ONE formatting guidelines and ensured that the manuscript conforms to the journal’s formatting and submission requirements.

4. We note that the grant information you provided in the ‘Funding Information’ and ‘Financial Disclosure’ sections do not match.

When you resubmit, please ensure that you provide the correct grant numbers for the awards you received for your study in the ‘Funding Information’ section.

Thank you for noting this discrepancy. The Funding Information and Financial Disclosure sections have now been revised to ensure consistency. The correct funding statement now reads:

This work was supported by Discovery Grants provided by the Natural Sciences and Engineering Research Council of Canada (EKC: RGPIN-2024-03946; GM: RGPIN-2022-03846). The funders had no role in study design, data collection and analysis, decision to publish, or preparation of the manuscript.

We have added this funding statement to the cover letter.

5. Thank you for stating the following financial disclosure:

“Sarvenaz Heirani Moghaddam received an Ontario graduate scholarship for this work. Drs. Erin Cressman and Gerome Manson's research are funded by NSERC’s Discovery Grant program.”

Please state what role the funders took in the study. If the funders had no role, please state: "The funders had no role in study design, data collection and analysis, decision to publish, or preparation of the manuscript."

If this statement is not correct you must amend it as needed.

Please include this amended Role of Funder statement in your cover letter; we will change the online submission form on your behalf.

Thank you for this comment. The funding statement has been updated to clarify the role of the funders. Specifically, we now state that the funders had no role in study design, data collection and analysis, decision to publish, or preparation of the manuscript.

6. Please expand the acronym “NSERC” (as indicated in your financial disclosure) so that it states the name of your funders in full.

This information should be included in your cover letter; we will change the online submission form on your behalf.

Thank you for this comment. The acronym NSERC has now been expanded to its full name, Natural Sciences and Engineering Research Council of Canada, in the financial disclosure statement.

7. Thank you for stating the following in your Competing Interests section:

“n/a”

Please complete your Competing Interests on the online submission form to state any Competing Interests. If you have no competing interests, please state "The authors have declared that no competing interests exist.", as detailed online in our guide for authors at http://journals.plos.org/plosone/s/submit-now

This information should be included in your cover letter; we will change the online submission form on your behalf.

Thank you for this comment. The Competing Interests statement has now been updated in the cover letter to read:

The authors have declared that no competing interests exist.

8. We note that Figure 1 in your submission contain copyrighted images. All PLOS content is published under the Creative Commons Attribution License (CC BY 4.0), which means that the manuscript, images, and Supporting Information files will be freely available online, and any third party is permitted to access, download, copy, distribute, and use these materials in any way, even commercially, with proper attribution. For more information, see our copyright guidelines: http://journals.plos.org/plosone/s/licenses-and-copyright.

We require you to either (1) present written permission from the copyright holder to publish these figures specifically under the CC BY 4.0 license, or (2) remove the figures from your submission:

1. You may seek permission from the original copyright holder of Figure 1 to publish the content specifically under the CC BY 4.0 license.

We recommend that you contact the original copyright holder with the Content Permission Form (http://journals.plos.org/plosone/s/file?id=7c09/content-permission-form.pdf) and the following text:

“I request permission for the open-access journal PLOS ONE to publish XXX under the Creative Commons Attribution License (CCAL) CC BY 4.0 (http://creativecommons.org/licenses/by/4.0/). Please be aware that this license allows unrestricted use and distribution, even commercially, by third parties. Please reply and provide explicit written permission to publish XXX under a CC BY license and complete the attached form.”

Please upload the completed Content Permission Form or other proof of granted permissions as an "Other" file with your submission.

In the figure caption of the copyrighted figure, please include the following text: “Reprinted from [ref] under a CC BY license, with permission from [name of publisher], original copyright [original copyright year].”

2. If you are unable to obtain permission from the original copyright holder to publish these figures under the CC BY 4.0 license or if the copyright holder’s requirements are incompatible with the CC BY 4.0 license, please either i) remove the figure or ii) supply a replacement figure that complies with the CC BY 4.0 license. Please check copyright information on all replacement figures and update the figure caption with source information. If applicable, please specify in the figure caption text when a figure is similar but not identical to the original image and is therefore for illustrative purposes only.

We have removed the photograph of the Kinarm from Figure 1.

9. Please remove your figures from within your manuscript file, leaving only the individual TIFF/EPS image files, uploaded separately. These will be automatically included in the reviewers’ PDF.

Thank you for this note. All figures have now been removed from the manuscript file. The figures have been uploaded separately as individual TIFF files in accordance with the PLOS ONE submission guidelines.

10. Please include captions for your Supporting Information files at the end of your manuscript, and update any in-text citations to match accordingly. Please see our Supporting Information guidelines for more information: http://journals.plos.org/plosone/s/supporting-information.

Thank you for this comment. Captions for all Supporting Information files have been added to the end of the main manuscript (after References). We have also reviewed and updated the in-text citations to ensure they correspond appropriately to the Supporting Information files.

11. We note that there is identifying data in the Supporting Information file < PLOS One Supporting Information.pdf>. Due to the inclusion of these potentially identifying data, we have removed this file from your file inventory. Prior to sharing human research participant data, authors should consult with an ethics committee to ensure data are shared in accordance with participant consent and all applicable local laws.

Data sharing should never compromise participant privacy. It is therefore not appropriate to publicly share personally identifiable data on human research participants. The following are examples of data that should not be shared:

-Name, initials, physical address

-Ages more specific than whole numbers

-Internet protocol (IP) address

-Specific dates (birth dates, death dates, examination dates, etc.)

-Contact information such as phone number or email address

-Location data

-ID numbers that seem specific (long numbers, include initials, titled “Hospital ID”) rather than random (small numbers in numerical order)

Data that are not directly identifying may also be inappropriate to share, as in combination they can become identifying. For example, data collected from a small group of participants, vulnerable populations, or private groups should not be shared if they involve indirect identifiers (such as sex, ethnicity, location, etc.) that may risk the identification of study participants.

Additional guidance on preparing raw data for publication can be found in our Data Policy (https://journals.plos.org/plosone/s/data-availability#loc-human-research-participant-data-and-other-sensitive-data) and in the following article: http://www.bmj.com/content/340/bmj.c181.long.

Please remove or anonymize all personal information (ID numbers that seem specific), ensure that the data shared are in accordance with participant consent, and re-upload a fully anonymized data set. Please note that spreadsheet columns with personal information must be removed and not hidden as all hidden columns will appear in the published file.

Thank you for bringing this to our attention and for removing the file from the submission while the issue was addressed. We have carefully reviewed the Supporting Information dataset and removed all potentially identifying information to ensure participant privacy is protected and that the data comply with PLOS ONE’s data-sharing policy for human research participants. Specifically, any variables that could directly or indirectly identify participants have been removed or anonymized. The revised dataset has been checked to ensure that no hidden columns or identifying metadata remain. The fully anonymized dataset has now been re-uploaded as Supporting Information.

12. If the reviewer comments include a recommendation to cite specific previously published works, please review and evaluate these publications to determine whether they are relevant and should be cited. There is no requirement to cite these works unless the editor has indicated otherwise.

Thank you for this note. We carefully evaluated all citation recommendations provided in the reviewer comments and included the suggested references where they were relevant to the present study.

Reviewer #1

Reviewer #1: The manuscript investigates whether implicit processes contribute to learning small mirror reversals compared to small visuomotor rotations. The study addresses a genuine gap in the literature. The finding that successful mirror reversal learners show no implicit aftereffects while rotation learners do appears robust for the specific subset of participants analysed (but see below). The manuscript is overall well written. However, there are fundamental concerns regarding participant exclusion and task comparability that require major revisions to ensure the soundness and validity of the interpretations. Please see below, thank you.

1. Participant exclusion

The most critical issue is the exclusion of 45% of the mirror reversal group (9/20 ppts) as "non-learners", compared to 0% in the VR group. The authors currently relegate this data to supplementary materials, but in reality, the difference in attrition rate is too substantial to be treated as a minor limitation. It fundamentally affects the interpretation of the results and the comparability of the tasks. Excluding nearly half of one experimental group while retaining all participants in the control group is at high risk of creating a selection bias. The authors should integrate the non-learner data into the main text rather than treating it as supplementary. This includes performing full-sample analyses that incorporate all 20 recruited participants in the mirror reversal group.

Moreover, the 3 SD learning criterion for inclusion appears arbitrary, and thus a sensitivity analysis would be a sensible approach to demonstrate how the results and conclusions change under different inclusion criteria (e.g., 2 SD, 2.5 SD, 4 SD). The goal here is to determine whether the absence of implicit learning is a robust feature of the task or instead an artefact of examining only the most successful learners.

We thank the reviewer for raising this important point. Because the present study was designed to examine the processes supporting successful MR learning, participants were initially classified based on whether they demonstrated learning to reach with the MR distortion or not. That said, we agree with the reviewer that it may appear that our approach introduced selection bias. We have thus now included the MR non-learners as an additional group in our analyses, reporting the results of a 4-group (MR-learners, MR-non-learners, VR-CW, VR-CCW) × 2 target (right, left) × 2 time (early trials, late trials) mixed ANOVA with repeated measures on the last two factors for variables of interest.

In the Data Analyses section, we have modified the following (P. 11, Line 265-276):

“The absolute values of the normalized AEs were compared between groups in a 4 group (MR-learner (MR-L), MR-non-learner (MR-NL), VR-CW, VR-CCW) x 2 target (right, left) x 2 time (early trials, late trials) mixed analysis of variance (ANOVA) with repeated measures (RM) on the last two factors. Participants who trained with VR CW and CCW rotations were considered to form separate groups to preserve target-wise compatibility with MR participants and to maintain comparable sample sizes across groups. We also compared AE variability and mean RT in the learning block across groups in a 4 group (MR-L, MR-NL, VR-CW, VR-CCW) x 2 target (right, left) x 2 time (early trials, late trials) mixed ANOVA with RM on the last two factors. A

Attachment Submitted filename: Response to Reviewers.pdf

# Peer review: Decision Letter 1

**DOI:** 10.1371/journal.pone.0333564.r003

**Authors:** Dimitris Voudouris

8 May 2026

PONE-D-25-49173R1

Implicit processes do not contribute to learning to reach in small mirror reversed visuomotor environments

PLOS One

Dear Dr. Heirani Moghaddam,

Thank you for submitting your manuscript to PLOS ONE. Both reviewers are satisfied with the revised version. Yet, they both raised some minor comments that should be addressed before a final decision is made. Please find these suggestions below.

Please submit your revised manuscript by Jun 22 2026 11:59PM. If you will need more time than this to complete your revisions, please reply to this message or contact the journal office at plosone@plos.org. When you're ready to submit your revision, log on to https://www.editorialmanager.com/pone/ and select the 'Submissions Needing Revision' folder to locate your manuscript file.

Please include the following items when submitting your revised manuscript:

- A letter that responds to each point raised by the academic editor and reviewer(s). You should upload this letter as a separate file labeled 'Response to Reviewers'.
- A marked-up copy of your manuscript that highlights changes made to the original version. You should upload this as a separate file labeled 'Revised Manuscript with Track Changes'.
- An unmarked version of your revised paper without tracked changes. You should upload this as a separate file labeled 'Manuscript'.

If you would like to make changes to your financial disclosure, please include your updated statement in your cover letter. Guidelines for resubmitting your figure files are available below the reviewer comments at the end of this letter.

If applicable, we recommend that you deposit your laboratory protocols in protocols.io to enhance the reproducibility of your results. Protocols.io assigns your protocol its own identifier (DOI) so that it can be cited independently in the future. For instructions see: https://journals.plos.org/plosone/s/submission-guidelines#loc-laboratory-protocols. Additionally, PLOS ONE offers an option for publishing peer-reviewed Lab Protocol articles, which describe protocols hosted on protocols.io. Read more information on sharing protocols at https://plos.org/protocols?utm_medium=editorial-email&utm_source=authorletters&utm_campaign=protocols.

As the corresponding author, your ORCID iD is verified in the submission system and will appear in the published article. PLOS supports the use of ORCID, and we encourage all coauthors to register for an ORCID iD and use it as well. Please encourage your coauthors to verify their ORCID iD within the submission system before final acceptance, as unverified ORCID iDs will not appear in the published article. *Only* the individual author can complete the verification step; PLOS staff *cannot* verify ORCID iDs on behalf of authors.

We look forward to receiving your revised manuscript.

Kind regards,

Dimitris Voudouris

Academic Editor

PLOS One

Journal Requirements:

1. If the reviewer comments include a recommendation to cite specific previously published works, please review and evaluate these publications to determine whether they are relevant and should be cited. There is no requirement to cite these works unless the editor has indicated otherwise.

2. Please review your reference list to ensure that it is complete and correct. If you have cited papers that have been retracted, please include the rationale for doing so in the manuscript text, or remove these references and replace them with relevant current references. Any changes to the reference list should be mentioned in the rebuttal letter that accompanies your revised manuscript. If you need to cite a retracted article, indicate the article’s retracted status in the References list and also include a citation and full reference for the retraction notice.

[Note: HTML markup is below. Please do not edit.]

Reviewer's Responses to Questions

**Comments to the Author**

1. If the authors have adequately addressed your comments raised in a previous round of review and you feel that this manuscript is now acceptable for publication, you may indicate that here to bypass the “Comments to the Author” section, enter your conflict of interest statement in the “Confidential to Editor” section, and submit your "Accept" recommendation.

Reviewer #1: (No Response)

Reviewer #2: (No Response)

**********

2. Is the manuscript technically sound, and do the data support the conclusions?

The manuscript must describe a technically sound piece of scientific research with data that supports the conclusions. Experiments must have been conducted rigorously, with appropriate controls, replication, and sample sizes. The conclusions must be drawn appropriately based on the data presented.

Reviewer #1: Yes

Reviewer #2: Yes

**********

3. Has the statistical analysis been performed appropriately and rigorously?

Reviewer #1: Yes

Reviewer #2: Yes

**********

4. Have the authors made all data underlying the findings in their manuscript fully available?

The PLOS Data policy requires authors to make all data underlying the findings described in their manuscript fully available without restriction, with rare exception (please refer to the Data Availability Statement in the manuscript PDF file). The data should be provided as part of the manuscript or its supporting information, or deposited to a public repository. For example, in addition to summary statistics, the data points behind means, medians and variance measures should be available. If there are restrictions on publicly sharing data—e.g. participant privacy or use of data from a third party—those must be specified.

Reviewer #1: Yes

Reviewer #2: Yes

**********

5. Is the manuscript presented in an intelligible fashion and written in standard English?

PLOS ONE does not copyedit accepted manuscripts, so the language in submitted articles must be clear, correct, and unambiguous. Any typographical or grammatical errors should be corrected at revision, so please note any specific errors here.

Reviewer #1: Yes

Reviewer #2: Yes

**********

6. Review Comments to the Author

Please use the space provided to explain your answers to the questions above. You may also include additional comments for the author, including concerns about dual publication, research ethics, or publication ethics. (Please upload your review as an attachment if it exceeds 20,000 characters)

Reviewer #1: I thank the authors for their thorough revision. I am happy to accept, with just one fifnal comment: the Bayes factors for the MR-L and MR-NL groups (BF₁₀ = 0.340 and BF₁₀ = 0.324, respectively) really correspond to anecdotal evidence for the null on standard scales (e.g., Lee & Wagenmakers, 2013), that is, just below the conventional threshold for moderate evidence. The authors may wish to relate the BF values to a standard evidence scale when describing the strength of support for the null

Reviewer #2: I would like to thank the authors for their thorough integration of my previous comments. I only have minor comments remaining.

AE Variability calculation: While I thank the authors for clarifying how they calculated angular variability, I still have some questions regarding this measure. Calculating variability by taking the SD over 24 trials may be a reasonable method for late learning, as participants have reached a plateau. However, during early learning, the SD doesn't necessarily tell us about the variability in reaching movements, since the mean is changing (as they are learning to adapt). This is further exemplified when stating that there was greater variability in early learning compared to late. This could simply be due to the fact that the AE values during early learning are much more spread out as the participant adapts. Is this a limitation in the variability measure? If so, consider identifying this limitation within the manuscript. Alternatively, other measures could be used to determine variability, such as performing a simple curve fit and using the mean residuals based on the fit as a measure of variability.

Figure 3A: It appears that the VR CW group is missing from figure 3A. Please amend this figure or clarify if any groups have been collapsed here in the figure caption.

**********

7. PLOS authors have the option to publish the peer review history of their article (what does this mean?). If published, this will include your full peer review and any attached files.

If you choose “no”, your identity will remain anonymous but your review may still be made public.

**Do you want your identity to be public for this peer review?** For information about this choice, including consent withdrawal, please see our Privacy Policy.

Reviewer #1: No

Reviewer #2: No

**********

[NOTE: If reviewer comments were submitted as an attachment file, they will be attached to this email and accessible via the submission site. Please log into your account, locate the manuscript record, and check for the action link "View Attachments". If this link does not appear, there are no attachment files.]

To ensure your figures meet our technical requirements, please review our figure guidelines: https://journals.plos.org/plosone/s/figures

You may also use PLOS’s free figure tool, NAAS, to help you prepare publication quality figures: https://journals.plos.org/plosone/s/figures#loc-tools-for-figure-preparation.

NAAS will assess whether your figures meet our technical requirements by comparing each figure against our figure specifications.

# Author comment: Author response to Decision Letter 2

**DOI:** 10.1371/journal.pone.0333564.r004

22 May 2026

Journal Requirements:

1. If the reviewer comments include a recommendation to cite specific previously published works, please review and evaluate these publications to determine whether they are relevant and should be cited. There is no requirement to cite these works unless the editor has indicated otherwise.

Thank you to the Editor for this note. We now site Lee & Wagenmakers, 2013 when reporting our Bayesian analysis results.

2. Please review your reference list to ensure that it is complete and correct. If you have cited papers that have been retracted, please include the rationale for doing so in the manuscript text, or remove these references and replace them with relevant current references. Any changes to the reference list should be mentioned in the rebuttal letter that accompanies your revised manuscript. If you need to cite a retracted article, indicate the article’s retracted status in the References list and also include a citation and full reference for the retraction notice.

We have updated our reference list to include all in-text citations. We have not cited any retracted articles.

Review Comments to the Author

Reviewer #1: I thank the authors for their thorough revision. I am happy to accept, with just one final comment: the Bayes factors for the MR-L and MR-NL groups (BF₁₀ = 0.340 and BF₁₀ = 0.324, respectively) really correspond to anecdotal evidence for the null on standard scales (e.g., Lee & Wagenmakers, 2013), that is, just below the conventional threshold for moderate evidence. The authors may wish to relate the BF values to a standard evidence scale when describing the strength of support for the null

Thank you to the Reviewer for this comment. We have updated the reporting of our Bayesian analysis to indicate “anecdotal-to-moderate” evidence in favor of the null hypothesis, in accordance wtih Lee & Wagenmakers, 2014. Specifically, we now state (P. 16, Line 381):

“Bayesian one sample t-tests indicated anecdotal-to-moderate support for the null hypothesis (23) in both the MR-L group and the MR-NL group (all p > 0.05; MR-L: BF₁₀ = 0.340; MR-NL: BF₁₀ = 0.324), suggesting that implicit learning was not significantly different from zero in either the MR-L or the MR-NL groups.”

Reviewer #2: I would like to thank the authors for their thorough integration of my previous comments. I only have minor comments remaining.

AE Variability calculation: While I thank the authors for clarifying how they calculated angular variability, I still have some questions regarding this measure. Calculating variability by taking the SD over 24 trials may be a reasonable method for late learning, as participants have reached a plateau. However, during early learning, the SD doesn't necessarily tell us about the variability in reaching movements, since the mean is changing (as they are learning to adapt). This is further exemplified when stating that there was greater variability in early learning compared to late. This could simply be due to the fact that the AE values during early learning are much more spread out as the participant adapts. Is this a limitation in the variability measure? If so, consider identifying this limitation within the manuscript. Alternatively, other measures could be used to determine variability, such as performing a simple curve fit and using the mean residuals based on the fit as a measure of variability.

Thank you to the Reviewer for this comment. As indicated, early adaptation would be associated with greater variability as there is great adjustment in participants’ movements as they look to compensate for the visuomotor distortion. We have now noted this limitation in our discussion. Furthermore, we indicate that variability continues to be higher for our MR participants compared to our VR participants even in late learning trials, suggesting that participants did not achieve a plateau and continued to adjust their movements. We have now added the following to our discussion (P. 19, Line 466):

“The increased movement variability observed in MR participants compared to VR participants may be a signature of the motor system’s attempt to explore and establish a new sensorimotor mapping under conditions where implicit learning mechanisms are not engaged. This increased variability was observed across both early and late learning trials (i.e., the standard deviation of the first 24 (early) and last 24 (late) learning trials), even though mean AE did not differ significantly between the MR-L and VR groups. We interpret our variability estimates during early learning cautiously, as participants were learning to reach with the MR or VR distortion and AE would be expected to change across trials. However, the sustained increase in variability even in late learning trials suggests performance by MR participants did not stabilize to the same extent as VR participants, and participants continued to adjust their reaches from trial to trial.”

Figure 3A: It appears that the VR CW group is missing from figure 3A. Please amend this figure or clarify if any groups have been collapsed here in the figure caption.

Thank you to the Reviewer for this comment. We have updated Figure 3A to include the VR CW group.

Attachment Submitted filename: Response to Reviewers.docx

# Editor report: Decision Letter 2

**DOI:** 10.1371/journal.pone.0333564.r005

**Authors:** Dimitris Voudouris

27 May 2026

Implicit processes do not contribute to learning to reach in small mirror reversed visuomotor environments

PONE-D-25-49173R2

Dear Dr. Heirani Moghaddam,

We’re pleased to inform you that your manuscript has been judged scientifically suitable for publication and will be formally accepted for publication once it meets all outstanding technical requirements.

Within one week, you’ll receive an e-mail detailing the required amendments. When these have been addressed, you’ll receive a formal acceptance letter and your manuscript will be scheduled for publication.

An invoice will be generated when your article is formally accepted. Please note, if your institution has a publishing partnership with PLOS and your article meets the relevant criteria, all or part of your publication costs will be covered. Please make sure your user information is up-to-date by logging into Editorial Manager at Editorial Manager® and clicking the ‘Update My Information' link at the top of the page. For questions related to billing, please contact billing support.

If your institution or institutions have a press office, please notify them about your upcoming paper to help maximize its impact. If they’ll be preparing press materials, please inform our press team as soon as possible -- no later than 48 hours after receiving the formal acceptance. Your manuscript will remain under strict press embargo until 2 pm Eastern Time on the date of publication. For more information, please contact onepress@plos.org.

Kind regards,

Dimitris Voudouris

Academic Editor

PLOS One

Additional Editor Comments (optional):

Reviewers' comments:

# Editor report: Acceptance letter

**DOI:** 10.1371/journal.pone.0333564.r006

**Authors:** Dimitris Voudouris

PONE-D-25-49173R2

PLOS One

Dear Dr. Heirani Moghaddam,

I'm pleased to inform you that your manuscript has been deemed suitable for publication in PLOS One. Congratulations! Your manuscript is now being handed over to our production team.

At this stage, our production department will prepare your paper for publication. This includes ensuring the following:

* All references, tables, and figures are properly cited

* All relevant supporting information is included in the manuscript submission,

* There are no issues that prevent the paper from being properly typeset

You will receive further instructions from the production team, including instructions on how to review your proof when it is ready. Please keep in mind that we are working through a large volume of accepted articles, so please give us a few days to review your paper and let you know the next and final steps.

Lastly, if your institution or institutions have a press office, please let them know about your upcoming paper now to help maximize its impact. If they'll be preparing press materials, please inform our press team within the next 48 hours. Your manuscript will remain under strict press embargo until 2 pm Eastern Time on the date of publication. For more information, please contact onepress@plos.org.

You will receive an invoice from PLOS for your publication fee after your manuscript has reached the completed accept phase. If you receive an email requesting payment before acceptance or for any other service, this may be a phishing scheme. Learn how to identify phishing emails and protect your accounts at https://explore.plos.org/phishing.

If we can help with anything else, please email us at customercare@plos.org.

Thank you for submitting your work to PLOS ONE and supporting open access.

Kind regards,

PLOS ONE Editorial Office Staff

on behalf of

Dr. Dimitris Voudouris

Academic Editor

PLOS One
